# Impact of organic and inorganic fertilizers on the yield and quality of silage corn intercropped with soybean

**DOI:** 10.7717/peerj.5280

**Published:** 2018-10-26

**Authors:** Ali Baghdadi, Ridzwan A. Halim, Ali Ghasemzadeh, Mohd Fauzi Ramlan, Siti Zaharah Sakimin

**Affiliations:** Department of Crop Science, Faculty of Agriculture, Universiti Putra Malaysia, Serdang, Selangor, Malaysia

**Keywords:** Corn silage, Fermentation analysis, Biological nitrogen fixation (BNF), Organic and inorganic fertilizer, Nutritive quality, Intercropping

## Abstract

**Background:**

Corn silage is an important feed for intense ruminant production, but the growth of corn relies heavily on the use of chemical fertilizers. Sustainable crop production requires careful management of all nutrient sources available on a farm, particularly in corn-based cropping systems.

**Methods:**

Experiments were conducted to determine the appropriate technique of corn-legume intercropping in conjunction with the supplemental use of chemical fertilizers, organic manure, and biofertilizers (BFs). Acetylene reduction assays (ARAs) were also performed on corn and soybean roots.

**Results:**

Combining chemical fertilizers with chicken manure (CM) in a 50:50 ratio and applying 50% NPK+50% CM+BF produced fresh forage and dry matter (DM) yields that were similar to those produced in the 100% nitrogen (N), phosphorus (P), potassium (K) treatment. Among the lone fertilizer treatments, the inorganic fertilizer (100% NPK) treatment produced the highest DM yield (13.86 t/ha) of forage and outyielded the 100% CM (9.74 t/ha) treatment. However, when CM was combined with NPK, the resulting DM yield of forage (13.86 t/ha) was the same as that resulting from 100% NPK (13.68 t/ha). Compared with CM applications alone, combinations of NPK and CM applications resulted in increased plant height, crop growth rates (CGRs) and leaf area index (LAI), but the values of these parameters were similar to those resulting from 100% NPK application. Fertilizers in which the ratio was 50% CM+50% NPK or 50% CM+50% NPK+BF resulted in protein yields that were similar to those resulting from conventional fertilizers. Similarly, the CP content did not significantly differ between applications of the 100% NPK and 50% CM+50% NPK fertilizers. The use of BFs had no significant impact on improving either the yield or quality of forage fertilized with inorganic or organic fertilizer. Lactic acid responded differently to different fertilizer applications and was significantly higher in the fertilized plots than in the unfertilized plots. Compared with treatments of lone chemical and lone organic manure fertilizers, treatments involving applications of BF and a combination of BF and NPK or CM resulted in higher ARA values.

**Discussion:**

There is no simple and easy approach to increase biological nitrogen fixation (BNF) in grain legumes grown as part of a cropping system under realistic farm field conditions. Overall, evidence recorded from this study proves that, compared with corn monocrops combined with CM and chemical fertilizers, corn-soybean intercrops could increase forage yields and quality, produce higher total protein yields, and reduce the need for protein supplements and chemical fertilizers.

## Introduction

Feed products from corn are characterized by a high-energy content but a relatively low crude protein content with low biological value (Summers, 2001; Mlynár, Rajčáková & Gallo, 2004). Corn forage, with its relatively high-energy content, is well adapted for use within low-cost rations for fattening livestock. Furthermore, corn forage can efficiently recycle plant nutrients, especially large amounts of nitrogen (N), phosphorus (P) and potassium (K) (Roth & Heinrichs, 2001).

Cereal and legume intercropping is important due to possible beneficial effects, including the improvement of forage quality via the complementary production of two or more crops grown concurrently in the same area (Eskandari, 2012a). Corn-legume intercropping can substantially increase forage quality and quantity and can reduce the need for protein supplementation (Ali & Mohammad, 2012). The yield of forage is an important part of grassland resources and describes the volume of dry matter (DM) available to livestock. Consequently, legume-cereal arrangements are considered a management practice for producing both forage quality and quantity (Eskandari, 2012b).

Corn productivity in tropical low-external input systems is usually limited by low soil fertility because crop uptake leads to a gradual depletion in soil nutrient stocks. Since the use of chemical fertilizers is undesirable, management of the fertility of these soils depends primarily on low-cost processes based on nutrient recycling (Figueiredo et al., 2009). The main processes that contribute to this recycling are biological nitrogen fixation (BNF) and nutrient recycling via organic fertilization. BNF can contribute to corn growth and yield by direct fixation within the corn plants or through the use of legume plants as crops either in rotation or intercropped with corn. BNF is usually considered a long-term sustainable N source for low-external input corn production systems (Figueiredo et al., 2009).

Being a fast-growing plant that performs C_4_ photosynthesis, corn requires a plentiful supply of N, P and K essential elements, which traditionally are obtained via inputs of chemical fertilizers to replenish soil N and P, resulting in high costs and environmental pollution (Dai et al., 2004; Awodun, 2007). The harmful effects on the environment due to the heavy use of N fertilizers are becoming more evident. There is a need for sustainable farming in which soil fertility is maintained by the use of inexpensive renewable resources that are readily available on the farm. The supply of other nutrients such as P can also be increased with the use of biofertilizers (BFs) (Mayer, Pfeiffer & Beyer, 2008; Oliveira et al., 2009). Organic fertilizers, including farmyard manure, chicken manure (CM), sheep manure, and BFs, may be used for crop production as a substitute for chemical fertilizers (Khan et al., 2005). Compared with chemical fertilizers, organic fertilizers improve soil fertility without leaving any residual effects in the soil and are much more inexpensive (Chater & Gasser, 1970).

Soybean plants are capable of supplying N for their own growth and for intercropped cereals via symbiotic N fixation and hence reduce the need for expensive and environmental-polluting N fertilizers (Rochester et al., 2001). However, additional studies are needed to fully understand the extent to which the N requirements of soybean grown at potential yield levels can be met by optimizing BNF alone as opposed to supplementing BNF with applied N (Salvagiotti et al., 2008).

Compared with monocropping systems, intercropping systems greatly contribute to crop production because of the effective use of resources (Inal et al., 2007). The major factor contributing to declining crop yields is reduced soil fertility caused by continuous cropping without the addition of sufficient mineral fertilizers and manure (Ndayisaba, 2014). The combined application of chemical and organic sources, which is usually referred to as integrated nutrient management, is widely recognized as a way of sustainably increasing crop productivity (Mahajan, Bhagat & Gupta, 2008).

The need to reduce costs of fertilizing crops with renewable forms of energy has revitalized the application of organic fertilizers worldwide. The main reason for promoting the increased use of organic materials is to improve environmental conditions and public health (Ojeniyi, 2000; Maritus & Vlelc, 2001).

Nutrients from organic manure are supplemented with inorganic nutrients that are readily available to plants (Ayoola & Makinde, 2008). Nutrients are released more slowly from organic manure and stored for longer time periods in the soil, thus ensuring a long residual effect (Sharma & Mittra, 1991; Abou el Magd, Hoda Mohammed & Fawz, 2005). CM was found to be a viable substitute for chemical fertilizers (Khan et al., 2005) and strongly positively affected both the moisture-holding capacity and structure of the soil (Sharif et al., 2004). Similarly, BFs could assist in increasing the efficiency of BNF via the production of plant growth-promoting substances (Kucey, Janzen & Leggett, 1989).

Integrated uses of fertilizer sources help maintain the fertility status of the soil. Applications of 75% NPK in combination with farmyard manure or phosphocompost in sorghum and soybean crops as well as 75% NPK in wheat caused wheat grain yields that were significantly higher than those in response to applications of inorganic and control fertilizers and saved 25% NPK fertilizer as a result of additional production by the following wheat crop (Ghosh et al., 2004).

However, the integration of modest amounts of inorganic fertilizers with organic materials such as CM, BFs and chemicals offers a strategy to meet smallholder crop nutrient requirements, especially in nutrient-intensive corn-legume intercropping systems. It seems that silage yield and quality are not reduced by intercropping even when NPK fertilizer application levels are reduced. Therefore, the objective of this experiment was to evaluate the impact of organic and/or inorganic fertilizer applications on the quality and quantity improvement of forage corn intercropped with soybean in relation to DM yield, nutritive value and volatile fatty acid (VFA) production.

## Materials and Methods

### Experimental site

The experiment was conducted in the agronomy fields of University Putra Malaysia, located at latitude 3:02′N longitude by 101:42′E on a sandy loam soil whose organic carbon content was 3.02% and whose pH was 6.03. The total annual rainfall in 2014 was approximately 2,689 mm; the monthly average was 224.08 mm. The rainfall peaks in November (333.1 mm), while the lowest occurs in June (139.4 mm). The mean monthly minimum and maximum temperatures range from 24.3 °C to 26.2 °C and from 31.5 °C to 35.1 °C, respectively, while the average monthly relative humidity ranges from 91.5 to 94.7%.

### Treatments and experimental design

The treatments consisted of ten different organic and inorganic fertilizer levels (a list of treatments is presented in Table 1). The intercrop composition was based on a replacement design. Each experimental unit was composed of eight crop rows, each 10 m in length. Each plot consisted of four rows of corn alternating with four rows of legumes, and the experiments were arranged as a randomized complete block design with four replications. A silage corn (*Zea mays*) cultivar (926) and a local soybean (*Glycine max*) cultivar were used in the current study.

**Table 1:** List of treatments.

Treatments	Description
T1	100% NPK or conventional fertilizer
T2	100% chicken manure (CM)
T3	Biofertilizer
T4	50% chicken manure + biofertilizer
T5	50% NPK + 50% chicken manure
T6	50% NPK+ biofertilizer
T7	50% NPK + 50% chicken manure + biofertilizer
T8	50% chicken manure
T9	50% NPK
T10	No fertilizer

**Notes.**

NPK nitrogen, phosphorus, potassium CM chicken manure BF biofertilizer

The BF preparation was conducted at the Soil Microbiology Laboratory, Department of Land Management, Universiti Putra Malaysia. The BF used in this study contained a consortium of N-fixing bacteria (SB13, SB16, SB26, SB35, SB42) and phosphate-solubilizing bacteria (PSB) (PSB16). For each bacterium, a 1 liter solution comprising 8 g of nutrient broth (NB) and 1 liter of distilled water was prepared in a flask. The flasks were autoclaved for 2 h and then transferred to a laminar flow hood (40 °C for 30 min). One to 2 full loops of 1 strain of bacteria were added to the flasks. All six flasks were placed on a shaker for 72 h (precipitation occurred), after which the contents were mixed and added to a single flask, which was then placed on a shaker for another 72 h. Seeds of corn and legumes (for BF treatments) were soaked in a BF solution for 90 min, after which the seeds were sown. After the seeds had germinated (1 week), the BF solution was applied to the BF treatments in the field. For a 5 liter BF solution, 4.9 liters of 2.5% molasses solution were prepared in an incubation tank, after which stock was added; the stock solution was then incubated for 2 days until the population reached 10^9^ cfu/ml (Panhwar et al., 2014). The BF spray volume was calculated on per-area basis (10 ml/plant). Based on the soil and CM analyses, the required level of CM was 10 t/ha, which was applied before planting. All other agronomic practices performed uniformly.

### Plant sampling, plant growth, yield and yield components

Corn and soybean intercrops were harvested concurrently. Corn was harvested when the kernel milk line was between 50 and 75%, and soybean was harvested at the seed-filling stage. The fresh weights of the harvested were obtained to determine fresh forage yield. The sampled area was 5 m^2^ in the center of each plot for the monocropped corn and intercropping treatments, and the fresh biomass weight was determined in grams per DM per square meter; aboveground plant parts were harvested by cutting the plants 2 cm above the soil surface by hand. Samples were oven dried at 70 °C for at least 72 h. Forage DM yields were calculated from the fresh and dry weights of the respective components listed above. Prior to harvest, measurements of parameters such as the leaf area index (LAI), photosynthesis and leaf chlorophyll content were taken; the equipment used included a LICOR LAI-2000 Plant Canopy Analyzer, LICOR LI-6400 Portable Photosynthesis System (Lincoln Nebraska USA), and a Minolta SPAD-502 chlorophyll meter, respectively.

### Nutritive quality measurements

Sample plants from each plot were cut into pieces, which were then mixed mechanically. Afterward, a 500 g subsample of each weighed forage sample was dried for 7 days in a 70 °C forced-air oven to constant moisture to determine forage quality characteristics. Dried subsamples were retained for forage quality assays. All dried samples were ground using a hammer mill to pass through a 1 mm screen and analyzed for their nutritive quality values. The quality of the forage samples was analyzed using near-infrared reflectance spectroscopy (NIRS) (Jafari, Connolly & Frolich, 2003). NIRS analyses involve exposing a sample (0.5–1.0 g) to an electromagnetic scan over a spectral wavelength range of 1,100 to 2,500 nm (near-infrared). Energy in this spectral range is directed onto the sample, and the reflected energy (R) is measured by the instrument. The diffuse reflection carries information that identifies chemical bonds within the sample, such as -CH, -OH, -NH and -SH bonds. The R is stored as the reciprocal logarithm (log 1/R), and the spectra are transformed to provide information about the chemical composition of the sample (Baker & Barnes, 1990; Shenk & Westerhaus, 1991).

### Determination of fermentation characteristics and ammonium-N

**Figure 1:**
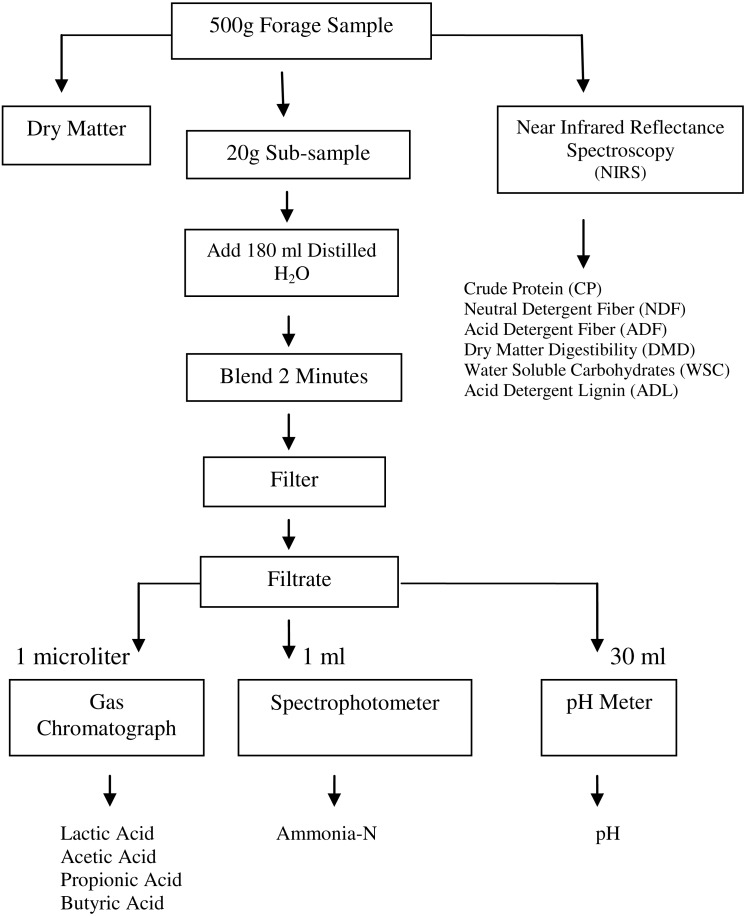
Fermentation analysis flow diagram.

Harvested forage material from each plot was cut into pieces with a forage chopper. After they were compacted via an Otosilager^^®^^ (forage-compacting machine), the forage was then ensiled in 20–25 liter plastic drums. The silos were stored in a laboratory, and their temperatures ranged from 25 to 30 °C (Baghdadi et al., 2016). After 10 weeks, the silos were opened, and representative samples were removed for analysis. Gas chromatography (GC) is the preferred method for measuring VFAs; therefore, VFAs were measured using an Agilent 7890A gas-liquid chromatograph (Agilent Technologies, Palo Alto, CA, USA) equipped with a flame ionization detector (FID). To determine VFAs and lactic acid, methyl n-valeric acid and fumaric acid were used as internal standards (Supelco, 1990; procedures cited from Lombard & Dowell, 1962). Figure 1 shows the process of the silage fermentation analysis. A pH electrode (Mettler-Toledo Ltd., England) was used to determine the pH of the silage after sample preparation. Ammonium-N was measured in corn-soybean silage samples via the colorimetric method described by Solorzano (1969).

### Determination of biological nitrogen fixation (BNF)

**Figure 2:**
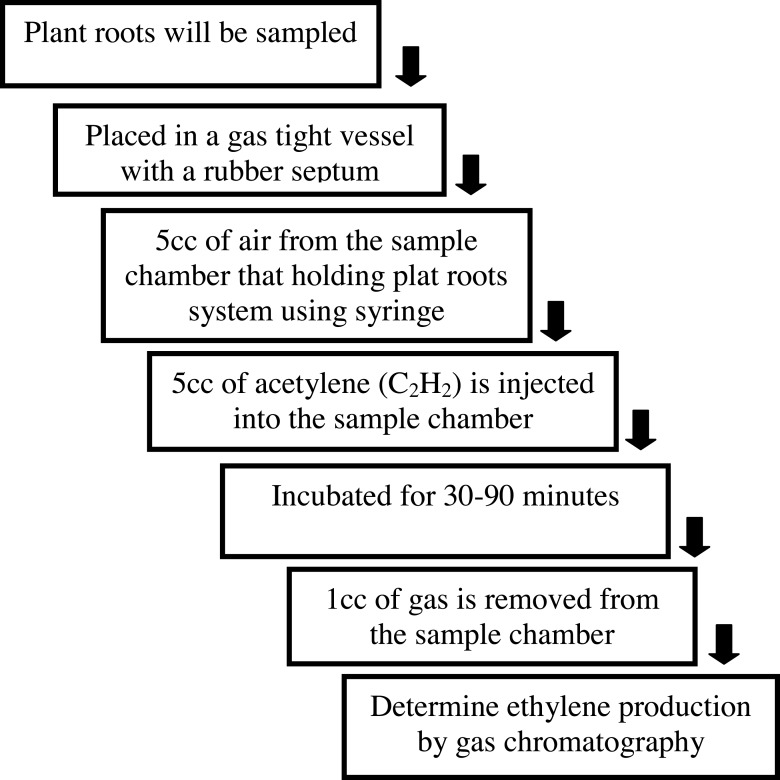
Acetylene reduction method to measure nitrogenase enzyme activity.

The discovery that the nitrogenase enzyme responsible for N fixation also reduces C_2_H_2_ (acetylene) to C_2_H_4_ (ethylene) Dilworth1966 provided a useful assay for the quantification of the N-fixation process. N fixation was estimated during vegetative growth (R_5_ stage of soybean—30 days after planting). Nodulated roots of soybean plants and roots of corn plants were placed in a 1,000 ml incubation vessel (polyvinyl chloride [PVC] wide-mouthed bottle). After the cap of the incubation vessel was secured tightly, 50 ml (5%) of the air was withdrawn from the incubation vessel (via a rubber septum in the cap) with a 50 ml plastic syringe; this air was replaced by 50 ml of acetylene. After one h of incubation, acetylene production was measured by injecting 1 ml of the headspace gas from each of the syringes into a gas chromatograph (Agilent 5890 Series Gas Chromatograph, Wilmington, DE, USA) equipped with a FID detector. Separation was achieved using an HP-Plot Q column (30 m × 0.53 mm × 40 m) (Agilent Technologies, Wilmington, DE, USA); N_2_ (99.9% purity, Domnick-Hunter generator, Domnick-Hunter, Leicester, UK) served as the carrier gas at a flow rate of 3.5 ml/min. An isothermal oven temperature of 50 °C was adopted for separation (Fig. 2). Calibration was completed with standard ethylene prepared by Scotty Specialty Gases (Supelco, Bellefonte, PA, USA). All the procedures were repeated three times. Ethylene produced by the nodules was calculated.

### Statistical analysis

The data were statistically processed using analysis of variance (ANOVA) with the Statistical Analysis Software (SAS) (Version 9.1). The least significant difference (LSD) was used to compare treatment means at the 0.01 and 0.05% probability levels.

## Results

### Yield and dry matter (DM) yield of corn-legume intercrops

**Figure 3:**
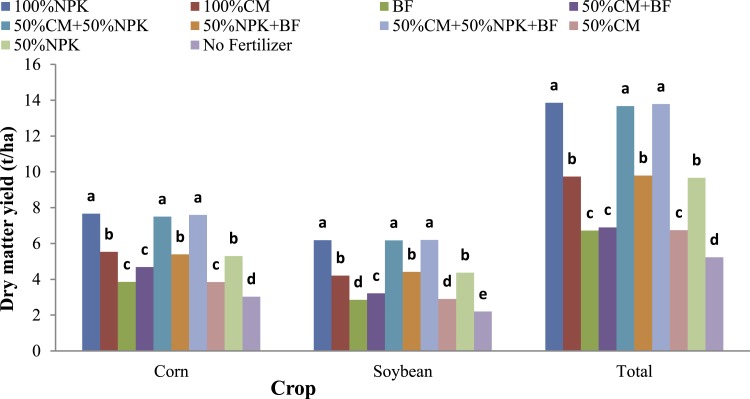
Forage dry matter (DM) yield of intercropped corn and soybean as influenced by integrated nutrient management. NPK, nitrogen, phosphorus, potassium; CM, chicken manure; BF, biofertilizer.

DM yield is a measure of forage productivity. Corn and soybean yields were higher than those of the control in all fertilizer treatments (*P* < 0.01). Among the three lone fertilizers, the 100% NPK fertilizer application resulted in significantly higher total forage DM yield (13.86 t/ha) than did the 100% CM fertilizer (9.74 t/ha) application, which was also significantly higher than that resulting from the BF (6.72 t/ha) application. Application of 50% NPK+50% CM resulted in similar DM yields (13.68 t/ha) as did application of 100% NPK, which implies that the CM can substitute for half of the NPK fertilizer without affecting DM yields. Application of 50% NPK+50% CM resulted in higher DM yields (13.68 t/ha) than did application of 100% CM fertilizer (9.74 t/ha) (Fig. 3).

No significant response of forage DM yield to BF application was observed for the combination of NPK and CM. Application of 50% NPK resulted in the same DM yield (9.67 t/ha) as that from 100% CM (9.74 t/ha). The DM yield from 100% NPK fertilizer application was significantly higher than that from 50% NPK fertilizer application. Similarly, the application of 100% CM fertilizer resulted in a significantly higher DM yield (9.74 t/ha) than did the application of 50% CM fertilizer (6.75 t/ha). These results showed that application of 50% NPK fertilizer or 50% CM alone could not fulfill the nutrient requirement of the plants. Compared with the control treatment, the combination of organic manure, chemicals and BF treatment resulted in higher forage DM yields, but the DM yields were similar to those resulting from the 100% NPK and the 50% NPK+50% CM treatments.

### Plant growth characteristics

Among the lone fertilizers, compared with 100% CM (184 cm), 100% NPK resulted in significantly taller corn plants (197 cm); the plants were also significantly taller than those resulting from the BF (152.50 cm) treatment. Similarly, compared with 100% CM (99 cm), 100% NPK resulted in taller soybean plants (109.25 cm), which were also significantly taller than those resulting from the BF (76.50 cm) treatment (*P* < 0.01) (Table 2).

**Table 2:** Plant growth parameters of intercropped corn and soybean as influenced by integrated nutrient management.

Treatments	Plant height corn(cm)	Plant height soybean(cm)	CGR(g/m^2^/day) corn	CGR(g/m^2^/day) soybean	LAI corn	LAI soybean
100%NPK	197.00a	109.25ab	29.34a	9.55a	2.56a	3.17a
100%CM	184.00b	99.00c	26.37b	8.48b	2.39b	3.07b
BF	152.50c	76.50d	22.51c	7.75c	2.15c	2.78c
50%CM+BF	152.00c	77.75d	22.40c	7.72c	2.12c	2.75c
50%CM+50%NPK	195.25a	112.75a	28.98a	9.39a	2.52a	3.20a
50%NPK+BF	185.00b	100.00c	26.98b	8.63b	2.37b	3.05b
50%CM+50%NPK+BF	195.00a	112.00a	29.23a	9.61a	2.54a	3.20a
50%CM	153.00c	78.00d	22.48c	7.78c	2.12c	2.78c
50%NPK	186.25b	99.75c	26.35b	8.55b	2.34b	3.02b
No Fertilizer	127.00d	64.00e	17.80d	7.26d	1.84d	2.54d
LSD(0.05)	3.49	2.20	1.002	0.40	0.04	0.05
*P* Value	<.0001	<.0001	<.0001	<.0001	<.0001	<.0001

**Notes.**

Mean values followed by the same letter in the same column are not significantly different at *P* < 0.05, based on least significant difference test (LSD).

NPK nitrogen, phosphorus, potassium CM chicken manure BF biofertilizer CGR crop growth rate LAI leaf area index

The integration of chemical fertilizer with CM increased corn and soybean plant heights to the same level as the heights in response to the inorganic fertilizer alone. Application of 50% NPK+50% CM resulted in corn plants that were as tall (195.25 cm) as those resulting from the lone NPK fertilizer application. Additionally, fertilizer treatments of 50% NPK+50% CM resulted in the same soybean plant heights (112.75 cm) as those resulting from the treatment of 100% NPK. Compared with that in response to 100% CM, the plant height of corn and soybean in response to combined applications of chemical fertilizers and CM was significantly taller.

Corn and soybean crop growth rates (CGRs) and the LAI in response to all fertilizer treatments were significantly higher than those in response to the control treatment (*P* < 0.01). A higher corn CGR was obtained with 100% NPK (29.34 g/m^2^/day) compared to both 100% CM (26.37 g/m^2^/day) and BF alone (22.51 g/m^2^/day). Similarly, among the three lone fertilizers, 100% NPK resulted in significantly higher soybean CGRs (9.55 g/m^2^ /day) than did 100% CM (8.48 g/m^2^/day), which was also resulted in CGRs that were significantly higher than those resulting from BF (7.75 g/m^2^/day).

Application of 50% NPK+50% CM yielded the same corn CGR (28.98 g/m^2^/day) as did application of NPK alone. Additionally, the fertilizer treatment of 50% NPK+50% CM yielded the same soybean CGR (9.39 g/m^2^/day) as did the fertilizer treatment of 100% NPK. The results showed that, compared with those in response to 100% CM, the CGR values of both crops in response to combined applications of NPK and CM were significantly higher.

Among lone fertilizer applications, 100% NPK resulted in the highest LAI among corn plants (2.56), followed by 100% CM (2.39) and BF (2.15). In addition, compared with CM alone (3.07), NPK alone resulted in a significantly higher soybean LAI (3.17), which was also significantly higher than that resulting from BF alone (2.78). The combination of NPK and CM fertilizers increased the corn and soybean LAI to the same level as that resulting from the NPK fertilizer alone. Application of 50% NPK+50% CM yielded the same corn LAI (2.52) as did application of NPK alone. Similarly, treatment with 50% NPK+50% CM yielded the same soybean LAI (3.20) as did treatment with 100% NPK. The corn and soybean LAI in response to the combined application of NPK and CM was significantly higher than that in response to application of 100% CM. Compared with the control treatment, the combination of BF, organic manure and chemical fertilizer treatment resulted in an increase in corn and soybean LAI, but the value was similar to that resulting from the 100% NPK and 50% NPK+50% CM treatments.

### Corn-legume forage nutritive quality

The results showed that the CP concentration of the corn-soybean forage was markedly affected by the different fertilizer treatments. All fertilizer application treatments yielded significantly higher mean CP percentages than did the control treatment (*P* < 0.01). Among the lone fertilizer applications, the mean CP percentage was not significantly different between the 100% NPK (14.48%) and 100% CM (14.50%) fertilizer applications, but those CP percentages were significantly higher than that in response to BF applications (13.02%) (Table 3).

**Table 3:** Forage nutritive quality of intercropped corn and soybean as influenced by integrated nutrient management.

Treatments	CP(%)	NDF(%)	ADF(%)	DMD(%)	WSC(%)	ADL(%)	Protein yield(Kg/ha)
100%NPK	14.48a	57.34a	35.68a	66.31	22.02	3.67	2,009.98a
100%CM	14.50a	49.22b	26.66b	65.69	22.16	3.69	1,408.95b
BF	13.02c	58.13a	36.09a	66.53	22.36	3.74	894.25d
50%CM+BF	13.38b	58.00a	36.17a	66.98	22.17	3.75	923.74d
50%CM+50%NPK	14.55a	49.67b	26.79b	66.73	22.15	3.62	1,991.95a
50%NPK+BF	13.45b	58.52a	36.12a	65.87	22.47	3.77	1,307.87c
50%CM+50%NPK+BF	14.56a	49.46b	26.74b	66.82	22.73	3.70	2,005.75a
50%CM	13.28b	57.39a	35.23a	66.95	22.43	3.80	896.03d
50%NPK	13.33b	57.73a	35.30a	66.92	22.70	3.74	1,288.39c
No Fertilizer	12.28d	57.93a	36.48a	66.36	22.21	3.76	642.62e
LSD(0.05)	0.33	1.64	1.84	ns	ns	ns	69.19
*P* Value	<.0001	<.0001	<.0001	0.3899	0.9919	0.9905	<.0001

**Notes.**

Mean values followed by the same letter in the same column are not significantly different at *P* < 0.05, based on least significant difference test (LSD).

NPK nitrogen, phosphorus, potassium CM chicken manure BF biofertilizer CP Crude protein NDF neutral detergent fiber ADF acid detergent fiber DMD dry matter digestibility WSC water soluble carbohydrates ADL acid detergent Lignin

The combination of chemical fertilizer and CM increased the mixed forage CP concentration to the same level as that in response to the chemical fertilizer alone. Application of 50% NPK+50% CM yielded the same CP content (14.55%) as did application of 100% NPK, which implies that CM can substitute for half of the NPK fertilizer to obtain good-quality forage without affecting DM yield. The integration of chemical fertilizer and CM increased the CP content to the same level as that in response to CM alone. Application of 50% NPK+50% CM yielded the same CP content (14.55%) as did application of 100% CM.

No significant response of mixed forage CP concentration to BF application was observed when integrated with NPK. Application of 50% NPK+BF yielded the same CP content (13.45%) as did application of 50% NPK (13.33%). Similarly, no significant response of forage CP content to BF application was observed when combined with CM. Treatment with 50% CM+BF did not increase the CP concentration to the same level as that in response to treatment with 100% CM. Application of 50% CM+BF yielded the same CP content (13.38%) as did application of 50% CM (13.28%). There were no positive effects of BF in combination with NPK or CM on forage CP concentrations.

The corn-soybean forage CP in response to 100% NPK fertilizer application was significantly higher than the CP in response to 50% NPK fertilizer application. Similarly, the fertilizer application of 100% CM yielded significantly higher CP than did the fertilizer application of 50% CM. The results showed that application of 50% NPK fertilizer or 50% CM fertilizer alone could not improve mixed forage quality because of the CP concentration. Compared with the control treatment, the integration of BF, organic manure and chemical fertilizer treatment resulted in increased forage CP concentration, but the concentration was similar to that resulting from the 50% NPK+50% CM and 100% NPK application treatments.

Among the lone fertilizer applications, the NDF concentration was not significantly different between the 100% NPK (57.34%) and lone BF (58.13%) fertilizer applications, but those NDF concentrations were significantly higher than that resulting from the CM fertilizer application alone (49.22%). Similarly, the ADF content was not significantly different between the 100% NPK (35.68%) and BF (36.09%) fertilizer applications, but those ADF contents were significantly higher than that resulting from the 100% CM fertilizer application (26.66%).

Application of 50% NPK+50% CM yielded a lower NDF content (49.67%) than did application of 100% NPK. In addition, the combination of 50% NPK+50% CM yielded a lower ADF content (26.79%) than that in response to 100% NPK. The combination of chemical fertilizer and CM reduced the NDF and ADF concentrations to the same levels as those in response to CM alone. Application of 50% NPK+50% CM yielded the same NDF (49.67%) and ADF concentrations (26.79%) as did application of 100% CM.

No significant response of forage NDF and ADF concentrations to BF applications was observed when integrated with NPK. Application of 50% NPK+BF resulted in the same NDF (58.52%) as did application of 50% NPK (57.73%). In addition, the combination of 50% NPK+BF resulted in the same ADF (36.12%) as that resulting from 50% NPK (35.30%). Similarly, no significant response of forage NDF and ADF contents to BF application was observed when combined with CM. Application of 50% CM+BF yielded the same NDF content (58%) as that yielded by application of 50% CM (57.39%). Additionally, treatment with 50% CM+BF yielded the same ADF content (36.17%) as did 50% CM (35.30%). There were no positive effects of BF in combination with NPK or CM on the forage concentrations of NDF and ADF.

Application of 50% NPK yielded the same NDF content (57.73%) and ADF content (35.30%) as did application of 100% NPK. In addition, the corn-soybean forage NDF content resulting from 100% CM (49.22%) fertilizer application was significantly lower than the NDF content resulting from 50% CM (57.39%) fertilizer application. Similarly, the application of 100% CM yielded significantly lower ADF content (26.66%) than did the application of 50% CM (35.23%). Compared with the control treatment, the combination of chemical, organic manure and BF treatment resulted in lower NDF and ADF concentrations, but those concentrations were similar to those resulting from the 100% CM and 50% NPK+50% CM treatments.

The results showed that the CP yield of corn-soybean forage was markedly affected by different fertilizer treatments (Tables 3, 4). Compared with the control treatment, all fertilizer application treatments resulted in a significantly higher mean CP yield (*P* < 0.01). Among the one fertilizer applications, the mean CP yield was not significantly different between the 100% NPK (2,009.98 kg/ha) and 100% CM (1,408.95 kg/ha) applications, but those values were significantly higher than those resulting from BF applications (894.25 kg/ha).

Integration of chemical fertilizer and CM increased the mixed forage CP yield to the same level as that in response to the chemical fertilizer alone. Application of 50% NPK+50% CM resulted in the same CP yield (1,991.95 kg/ha) as did application of 100% NPK, which implies that CM can substitute for half of NPK to produce high protein-yielding forage without affecting DM yields. Application of 50% NPK+50% CM fertilizer resulted in higher CP yields (1,991.95 kg/ha) than did application of 100% CM fertilizer (1,408.95 kg/ha).

No significant response of forage CP yield to BF application combined with NPK was observed. Application of 50% NPK+BF resulted in the same CP yield (1,307.87 kg/ha) as did application of 50% NPK (1,288.39 kg/ha). Similarly, no significant response of forage CP yield to BF application combined with CM was observed. Treatment with 50% CM+BF did not increase CP yields to the same levels as those in response to treatment with 100% CM. Application of 50% CM+BF resulted in the same CP yield (923.74 kg/ha) as did application of 50% CM (896.03 kg/ha). No positive effects of BF in combination with NPK or CM on forage CP yields were observed.

The corn-soybean forage CP yield in response to 100% NPK fertilizer application (2,009.98 kg/ha) was significantly higher than the CP yield in response to 50% NPK fertilizer application (1,288.39 kg/ha). Similarly, application of 100% CM resulted in significantly higher CP yield (1,408.95 kg/ha) than did application of 50% CM (896.03 kg/ha). The results showed that application of 50% NPK fertilizer or 50% CM fertilizer alone could not fulfill the nutrient requirements of plants with respect to CP yield production. Compared with the control treatment, the combination of organic manure, chemical and BF treatment resulted in increased forage CP yields, but the values were similar to those resulting from the 100% NPK and 50% NPK+50% CM treatments.

The effects of chemical fertilizers, CM, and BF as well as their combinations were not significant for WSC concentrations; the WSC concentration in the forage material ranged from 22.02 to 22.73%. Additionally, the effects of different fertilizer application treatments were not significant for DMD, which ranged from 65.69 to 66.98%. Furthermore, the effects of lone and combined fertilizer treatments were not significant for ADL concentrations; the ADL concentration in the forage material ranged from 3.62 to 3.80%.

### Volatile fatty acids (VFAs) in silage

With respect to the lone fertilizer applications, the lactic acid content was not significantly different between the 100% NPK (4.04%) and sole CM (4.02%) fertilizer applications, but those contents were significantly higher than those resulting from BF application alone (3.91%). Similarly, the ammonia-N content did not significantly differ in response to 100% NPK (2.06%) or 100% CM (2.07%) treatments, but those percentages were significantly higher than the percentage resulting from the BF (1.84%) treatment (Table  4).

**Table 4:** Silage volatile fatty acids (VFA) of intercropped corn and soybean as influenced by integrated nutrient management.

Treatment	pH	DM range(%)	Lactic acid(% of DM)	Acetic acid(% of DM)	Propionic acid(% of DM)	Butyric acid(% of DM)	Ammonia N(% of DM)
100%NPK	4.02	32.61	4.04a	1.62	0.16	0.03	2.06a
100%CM	4.03	32.24	4.02ab	1.63	0.16	0.03	2.07a
BF	4.02	32.52	3.91d	1.63	0.15	0.04	1.84b
50%CM+BF	4.03	32.49	3.92cd	1.63	0.16	0.03	1.83b
50%CM+50%NPK	4.03	32.77	4.01abc	1.62	0.16	0.03	2.08a
50%NPK+BF	4.03	32.33	3.93bcd	1.64	0.15	0.03	1.83b
50%CM+50%NPK+BF	4.02	32.78	4.03a	1.63	0.14	0.03	2.04a
50%CM	4.02	32.39	3.92cd	1.63	0.14	0.03	1.82b
50%NPK	4.02	32.25	3.92cd	1.62	0.15	0.03	1.83b
No Fertilizer	4.03	32.44	3.89d	1.64	0.15	0.04	1.81b
LSD(0.05)	ns	ns	0.10	ns	ns	ns	0.19
*P* Value	0.9999	0.6425	0.0120	0.9992	0.9246	0.9532	0.0065

**Notes.**

Mean values followed by the same letter in the same column are not significantly different at *P* < 0.05, based on least significant difference test (LSD).

NPK nitrogen, phosphorus, potassium CM chicken manure BF biofertilizer DM dry matter

Integration of NPK and CM increased mixed silage lactic acid and ammonia-N concentrations to the same levels as those resulting from chemical fertilizer. Treatment with 50% NPK+50% CM yielded the same lactic acid content (4.01%) as did treatment with 100% NPK. Likewise, application of 50% NPK+50% CM yielded the same ammonia-N concentration (2.08%) as did application of 100% NPK application. The combination of chemical fertilizer and CM increased lactic acid and ammonia-N contents to the same levels as those resulting from CM fertilizer. Application of 50% NPK+50% CM yielded the same lactic acid content (4.01%) as did application of 100% CM. In addition, application of 50% NPK+50% CM yielded the same ammonia-N concentration as did application of 100% NPK.

No significant response of silage lactic acid and ammonia-N contents to BF application combined with chemical fertilizer was observed. Treatment with 50% NPK+BF yielded the same lactic acid content (3.93%) as did treatment with 50% NPK (3.92%). Additionally, application of 50% NPK+BF yielded the same ammonia-N content (1.83%) as did application of 50% NPK (1.83%).

No significant response of silage lactic acid and ammonia-N contents to BF application combined with CM was observed. Application of 50% CM+BF yielded the same lactic acid content (3.92%) as did application of 50% CM (3.92%). Additionally, application of 50% CM+BF yielded the same ammonia-N content (1.83%) as did application of 50% CM (1.83%) treatment. No positive effects of BF in combination with NPK or CM were observed on silage lactic acid or ammonia-N contents. Silage lactic acid and ammonia-N contents resulting from 100% NPK were significantly higher than those resulting from 50% NPK. Similarly, the application of 100% CM yielded significantly higher lactic acid and ammonia-N contents than did application of 50% CM. Compared with the control treatment, the combination of NPK, organic manure and BF treatment resulted in increased silage lactic acid and ammonia-N contents, but those contents were similar to those resulting from the 50% NPK+50% CM and 100% NPK treatments.

The chemical fertilizers, CM, and BF as well as their combinations did not significantly affect the pH, which ranged from 4.02 to 4.03. In addition, the different fertilizer application treatments did not significantly affect the silage DM, which ranged from 32.24% to 32.78%. Furthermore, the lone and combination fertilizer treatments did not significantly affect acetic acid, propionic acid or butyric acid concentrations.

### Biological nitrogen fixation (BNF)

**Figure 4:**
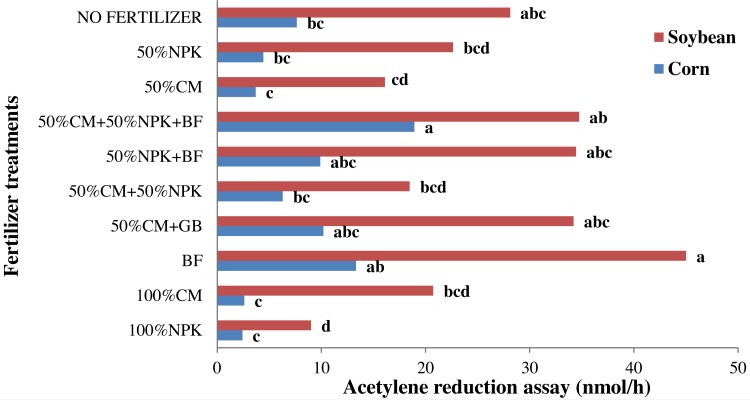
Effect of chemical, organic and biofertilizer on acetylene reduction assay (ARA) of corn and soybean roots. NPK, nitrogen, phosphorus, potassium; CM, chicken manure; BF, biofertilizer.

Fertilizer treatments significantly affected the results of the acetylene reduction assays (ARAs) of the corn and soybean roots. Compared with those in the chemical fertilizer treatment, the ARA rates of the corn roots in the BF treatment responded well (*P* < 0.05). Reducing the level of N in the fertilizer treatments led to significant increases in ARA rates. The ARA rates in the BF treatment were generally higher than those in the other lone fertilizer treatments. Among the lone fertilizer treatments, significant differences between 100% NPK, 100% CM and BF applications were recorded. The BF treatment yielded a higher ARA rate (13.33 nmol/h) than did the 100% CM (2.61 nmol/h) and 100% NPK (2.42 nmol/h) treatments. However, no significant differences were observed between the 100% NPK and 100% CM fertilizer treatments (*P* < 0.05), while the control treatment yielded a significantly higher ARA rate (7.63 nmol/h) than did the lone chemical and CM fertilizer treatments (Fig. 4).

The combined use of BF with NPK or CM resulted in increased ARA rates. Treatments with BF yielded higher ARA rates, but there were no significant differences among BF treatments. The application of BF alone yielded ARA rates similar to those yielded by 50% CM+50% NPK+BF (18.94 nmol/h), 50% CM+BF (10.19 nmol/h) and 50% NPK+BF (9.89 nmol/h) fertilizers (*P* > 0.05).

Compared with those in response to 100% CM and 100% NPK applications, the ARA rates in response to BF applications in the soybean roots responded well (*P* < 0.05). Among the lone fertilizers, significant differences between 100% NPK, 100% CM and BF applications were observed. The BF application showed a higher ARA rate (45.02 nmol/h) than did the 100% CM (20.74 nmol/h) and 100% NPK (9.01 nmol/h) applications. However, significant differences were not observed between the 100% NPK and 100% CM treatments (*P* < 0.05), while the control treatment yielded a significantly higher ARA value (28.12 nmol/h) than did the lone chemical and CM fertilizers. The ARA rates did not significantly different between the BF and unfertilized plots.

The ARA rate in response to BF (45.02 nmol/h) did not significantly differ from that in response to 50% CM+50% NPK+BF (34.74 nmol/h), 50% NPK+BF (34.46 nmol/h) or 50% CM+BF (34.22 nmol/h) (*P* < 0.05). In this experiment, BF application (alone or in combination with other fertilizers) significantly affected the ARA rates. Treatments involving all rates of BF yielded higher ARA values, but there were no significant differences in ARA values between the BF and control treatments.

## Discussion

The results showed that application of 100% NPK fertilizer was best in terms of yield and quality of the mixed forage of corn and soybean and was better than the 100% CM fertilizer. However, the combination of 50% NPK+50% CM produced similar results, which implies that CM can substitute for half of NPK without affecting the DM yield of forage. BF did not provide any benefits to forage yield and quality, alone or in combination with NPK or CM; therefore, application of BFs is not recommended.

Short plant height was due to the depletion of nutrients from the control plots over time; hence, plants exhibited stunted growth due to an inadequate supply of nutrients. The relatively taller plant height might be attributed to the gradual release of essential nutrients from the chemical and CM fertilizers as needed by the corn plant. The results of this experiment confirm the findings of Gonzalez, Alvarez & Matheus (2001) who reported that chemical fertilizer and organic manure, which were supplied as essential nutrition at the initial establishment stage, produced the best results for the measured parameters, such as plant height. Increased plant height in response to applications of combined fertilizer is attributed to more availability of N from both urea and manure throughout the growing season. These results are in agreement with the findings reported by Mitchell & Tu (2005) and Saleem (2010), who observed that, compared with control treatments, 50% poultry manure+50% chemical treatments resulted in the tallest corn plants in corn-legume cropping systems.

Applications of NPK and the combined use of organic and chemical fertilizers enhanced corn-soybean forage productivity and yield by increasing the LAI and CGR. As a cereal crop, corn responded well to exposure of its leaves to light, and its uptake of essential nutrients might be attributed to the synergistic action of fertilizer and organic amendments. These increases in plant growth traits were more evident when CM was combined with NPK fertilizer. Corn has a determinate growth habit, and yield is determined at the early growth stage. Plant availability of micro- and macronutrients at the vegetative stage is important, and this availability was provided by the faster release of nutrients from inorganic fertilizer than from organic fertilizers. Thus, 100% NPK resulted in better yields than did 100% CM, but when the two were combined, the yield was similar to that of 100% NPK.

The findings of this experiment are similar to those of Khan et al. (2009), who reported that, compared with applications of organic fertilizer alone, fertilizer applications of N combined with farmyard manure resulted in greater plant height, a larger LAI and greater biological yields of corn. The increased LAI observed in response to applications of organic and inorganic fertilizer combinations resulted in increased CGRs (Naing et al., 2010). The results were in agreement with observations made by Lelei, Onwonga & Freyer (2009), Saleem (2010), Ibeawuchi et al. (2007) and Khan et al. (2009), who reported that higher LAI and CGR values and higher biological yields of corn occurred in response to combinations of fertilizers than to lone fertilizers.

The growth of corn plants in response to combinations of chemical+organic fertilizers and lone inorganic fertilizers was comparable because nutrients were released early from the inorganic fertilizer, and corn, which is an aggressive feeder, was able to use those nutrients for growth. Although the rate of application of inorganic fertilizer was reduced in the combined use, complementation with nutrients from organic manure made possible yields that were comparable to those resulting from inorganic fertilizer applications alone. Chung et al. (2000) also reported that organic manure applications supplemented with an adequate amount of inorganic fertilizer resulted in relatively high DM yields of corn.

The use of chemical fertilizer instead of organic manure has some importance because the former readily supplies nutrients to crops, which helps increase both growth and yield (Meng, Ding & Cai, 2005; Manna et al., 2005). CM is also a rich source of nutrients that help improve crop yields; lower yields resulting from CM rather than from chemical fertilizer might be due to the slow release of nutrients. The yield increase in response to 50% NPK+50% CM application might be due to high levels of microbial activity, which might have enhanced organic matter decomposition as well as the release of plant-available nutrients.

In addition to supplying nutrients, organic manure also improves soil structure. In the present study, organic manure applications enhanced soil organic matter and soil nutrients, which were released slowly and steadily and were efficiently used during later growth stages of corn. The optimum yield obtained was partly attributable to the integration of organic and inorganic fertilizers: nutrients were released from chemical fertilizers, and corn was able to use those nutrients for growth, which was supplemented by necessary nutrients released from the decomposition of added organic manure. These results confirmed those of studies by other authors, e.g., Adeniyan & Ojeniyi (2005), Shata, Mahmoud & Siam (2007), Sial et al. (2007), Rehman, Bukhsh & Ishaque (2008), and Law-Ogbomo & Remison (2009). Vanlauwe et al. (2002) reported that the integrated use of inorganic and organic nutrient sources resulted in synergy and improved the conservation and synchronization of nutrient release and crop demand, which led to increased fertilizer efficiency and greater yields. Dudal & Roy (1995) indicated that the use of organic fertilizer could increase the efficiency of inorganic fertilizer.

While some research has indicated that increased amounts of chemical fertilizers may suppress soybean yields (Hiebsch & McCollum, 1987), in the present study, the positive response of corn to NPK (Jahanzad et al., 2014) compensated for the probable yield suppression of soybean, which ultimately resulted in the greatest forage DM yield in the 100% NPK and 50% CM+50% NPK treatments.

Chemical fertilizers are equally rich in all three essential nutrients. However, organic fertilizers may be rich in one of the three nutrients or may have low levels of all three nutrients. Chemical fertilizers are always available to provide an immediate supply of nutrients to plants if the situation demands. In contrast to inorganic fertilizers, one aspect of organic fertilizers is their slow-release capability. Slow release means there is less risk of over-fertilization, but sometimes the slow release of organic fertilizers is not able to fulfill the supply of the nutrients needed when required.

N is present in manure in a variety of forms, most of which is gradually converted to ammonium-N and nitrate-N. Regardless of the chemical fertilizer, the same form is ultimately available to plants. Organic fertilizers such as manure add nutrients to the soil, which increases soil organic matter, improves soil structure and improves buffering capacity against fluctuations in pH levels.

Compared with chemical fertilizers, lone organic fertilizers or BFs do not meet the nutritional needs of crops because they contain a comparatively less quantity of nutrients. The results of this study showed that the DM yield from the combination of inorganic and organic fertilizers was higher than that from the lone application of organic fertilizer or BF. Makinde, Agboola & Oluwatoyinbo (2001) indicated that corn performance resulting from chemical fertilizer applications alone and a mixture of organic and inorganic fertilizer applications was significantly higher than that resulting from organic fertilizer application alone. Similarly, Murwira & Kirchmann (1993) reported that the nutrient use efficiency of a crop can increase in response to an integrated application of mineral fertilizer and organic manure.

Satyanarayana et al. (2002) reported an optimum grain yield of rice with an application of 10 t/ha farmyard manure supplemented with 120 kg N compared to manure alone and inorganic fertilizers alone. This result was attributed to increased nutrient uptake and increased numbers of tillers and filled grains per panicle. Combinations of CM and NPK fertilizers could therefore be considered a better option for increasing fertilizer use efficiency and providing a more balanced supply of nutrients (Suge, Omunyin & Omami, 2011).

In the present study, the results showed that BF application did not significantly increase the DM yield of forage. Compared with applications of combined NPK and CM fertilizers, applications of BF did not significantly increase DM yields. However, BFs could possess insufficient amounts of nutrients, which can alter the way plants grow (Lodwig & Poole, 2003).

BFs are different from chemical fertilizers and organic manure with respect to their effects on crop productivity. BFs affect the fixation of N or enhance the availability of nutrients, whereas chemical fertilizers directly provide nutrients to plants at a rapid speed. The nutrient release rate in response to BFs is too slow to meet crop requirements in a short time; hence, some nutrient deficiencies may occur. BFs maintain or increase soil microbial complexes by the slow release of mineral nutrients from organic matter over a long period of time; BFs do not directly supply any nutrients to crops. The major plant nutrients may not exist in sufficient quantities within BFs to sustain maximum crop growth (Chen, 2006). BFs are comparatively low in nutrient content, so a larger volume is needed to provide enough nutrients for crop growth (Chen, 2006; Carvajal-Muñoz & Carmona-Garcia, 2012). There is a gap in BF application timing and nutrient requirements for crops because the field performance of applied BFs depends on the fertility status of the field soil, the type of crop and the method of cultivation. Shortages of particular strains of microorganisms or nonideal growing conditions can reduce the availability of some biofertilizers.

The quality traits of forage significantly improved in response to applications of fertilizer. Compared with those in response to applications of lone BF and control treatments, forage DM yield and protein yield in response to 100% NPK, CM alone and NPK and CM combination treatments increased. These results were in agreement with the findings of Abbasi, Rouzbehan & Rezaei (2012), who reported that, compared with those in response to no fertilizer applications, corn-amaranth forage yield and the concentration of CP in response to fertilizer applications significantly increased.

Likewise, the CP content significantly increased in response to chemical fertilizers and the combination of inorganic and CM fertilizers. These results coincided with findings of some studies (Soto, Jahn & Arredondo, 2004). N contributes greatly to the synthesis of amino acids and ultimately proteins; the greater the available N to crops is; the greater the protein that can be synthesized. High levels of CP as a result of NPK applications, which leads to increased protein synthesis, have been reported in sorghum-lima bean forage (Zandvakili et al., 2012) and forage amaranth production (Abbasi, Rouzbehan & Rezaei, 2012).

The application of 100% NPK fertilizer increased the CP concentration but also increased the NDF and ADF concentrations. However, when combined with CM, 100% NPK resulted in lower NDF and ADF concentrations. These results confirm previous reports of Abbasi, Rouzbehan & Rezaei (2012), who reported increased NDF contents in forage amaranth in response to chemical fertilizer applications. Some researchers have reported meaningful results indicating that organic fertilization reduced ADF concentrations in corn production (Keskin et al., 2005). In the present study, organic manure fertilizer significantly reduced the NDF concentration, and compared with the other treatments, the 100% CM and CM combined with NPK or BF treatments yielded lower NDF and ADF concentrations.

Fertilizer application influenced some fermentation characteristics of corn-soybean silage. Lactic acid responded differently to different fertilizer applications; lactic acid was significantly higher in the fertilized plots than in the unfertilized plots. Generally, the presence of high levels of lactic acid suggests efficient fermentation and minimal DM loss of silage (Seglar, 2003). According to Seglar (2003), quality silage should contain <1 g/kg butyric acid, and elevated levels may cause silage deterioration due to secondary fermentation; however, acetic acid, propionic acid and butyric acid contents are not influenced by fertilizer applications.

Increased concentrations of ammonia-N result from the hydrolysis of proteins and adversely affect silage quality (Kung & Shaver, 2001). In the present study, these criteria characterize the silage produced in the intercropping system in response to applications of NPK and CM fertilizers and their combined use as good silage.

A chemical fertilizer is defined as any inorganic material of wholly or partially synthetic origin that is added to the soil to sustain plant growth. Many artificial fertilizers contain acids such as sulfuric acid and hydrochloric acid; these acids tend to increase the acidity of the soil, reducing the soil’s population of beneficial organisms and interfering with plant growth. In contrast, organic fertilizers support the growth of N-fixing bacteria. Generally, healthy soils contain enough N-fixing bacteria to fix sufficient atmospheric N to supply the needs of growing plants. However, the continued use of chemical fertilizers may diminish the amount of these N-fixing bacteria.

In summary, compared with 100% NPK and 100% CM fertilizers, no fertilizer, BF and combinations of BF with NPK or CM fertilizers tended to exhibit higher rates of acetylene reduction. No significant differences between the BF, combination and control treatments were observed in terms of ARA rates. Treatments that supply high levels of N exhibited lower values of ARA, and the maximum ARA rate was recorded in treatments that presented low levels of N.

## Conclusion

Among the fertilizer treatments in this experiment, the effects of conventional 100% NPK and 50% CM+50% NPK fertilizers were similar; these fertilizers significantly increased corn-soybean DM yields and improved the plant growth characteristics, forage quality, CP concentration, protein yield and quality of corn-soybean silage. CM has become one of the most important manures because it contains a relatively high N content. Compared with expensive chemical fertilizers, CM is locally available; hence, smallholding farmers can easily afford it, and manure is environmentally friendly. Complementary application of chemical fertilizers and organic manure can be a fertilizer management strategy in corn+legume intercropping systems. BF application (alone or combination with other fertilizers) had no significant benefit to forage DM yields or nutritive quality. The integrated application of CM and chemical fertilizers may be practiced to achieve proper productivity and quality of corn-soybean silage.

Increasing the level of N (100% NPK or 100% CM) led to a significant decrease in ARA rates. Management practices that increase N demand by the host plant are promising avenues for increasing N fixation in grain legumes in cropping systems.

##  Supplemental Information

10.7717/peerj.5280/supp-1 Supplemental Information 1 Determination of ethylene production by gas chromatography Raw data.

10.7717/peerj.5280/supp-2 Supplemental Information 2 Forage nutritive quality

10.7717/peerj.5280/supp-3 Supplemental Information 3 Silage volatile fatty acids (VFA)

10.7717/peerj.5280/supp-4 Supplemental Information 4 SAS final results

10.7717/peerj.5280/supp-5 Supplemental Information 5 Report ARA chromatogram

## Additional Information and Declarations

### Data Availability

The following information was supplied regarding data availability: The raw data are provided in the Supplemental Files.
