# Supplementary material for: Impact of organic and inorganic fertilizers on the yield and quality of silage corn intercropped with soybean
Source: PeerJ. 2018 Oct 26;6:e5280. doi: 10.7717/peerj.5280 (PMC6204818; doi:10.7717/peerj.5280)
Supplement: Supplemental Information 4 [file peerj-06-5280-s004.pdf]

The ANOVA Procedure

Class Level Information

Class	Levels	Values
Block	4	1 2 3 4
Fertilizer	10	T1 T10 T2 T3 T4 T5 T6 T7 T8 T9

Number of Observations Read 40
Number of Observations Used 40

The ANOVA Procedure

Dependent Variable: CPmix

Source	DF	Sum of Squares	Mean Square	F Value	Pr > F
Model	12	21.97631000	1.83135917	36.09	<.0001
Error	27	1.37008750	0.05074398		
Corrected Total	39	23.34639750			

R-Square	Coeff Var	Root MSE	CPmix Mean
0.941315	1.643935	0.225264	13.70275

Source	DF	Anova SS	Mean Square	F Value	Pr > F
Block	3	0.38048750	0.12682917	2.50	0.0808
Fertilizer	9	21.59582250	2.39953583	47.29	<.0001

The ANOVA Procedure

Dependent Variable: NDFmix

Source	DF	Sum of Squares	Mean Square	F Value	Pr > F
Model	12	603.9599600	50.3299967	39.28	<.0001
Error	27	34.5979175	1.2814044		
Corrected Total	39	638.5578775			

R-Square	Coeff Var	Root MSE	NDFmix Mean
0.945819	2.045549	1.131991	55.33925

Source	DF	Anova SS	Mean Square	F Value	Pr > F
Block	3	4.8068075	1.6022692	1.25	0.3111
Fertilizer	9	599.1531525	66.5725725	51.95	<.0001

The ANOVA Procedure

Dependent Variable: ADFmix

Source	DF	Sum of Squares	Mean Square	F Value	Pr > F
Model	12	710.0899300	59.1741608	36.78	<.0001
Error	27	43.4400675	1.6088914		
Corrected Total	39	753.5299975			

R-Square	Coeff Var	Root MSE	ADFmix Mean
0.942351	3.829166	1.268421	33.12525

Source	DF	Anova SS	Mean Square	F Value	Pr > F
Block	3	2.9483075	0.9827692	0.61	0.6138
Fertilizer	9	707.1416225	78.5712914	48.84	<.0001

The ANOVA Procedure

Dependent Variable: DMDmix

Source	DF	Sum of Squares	Mean Square	F Value	Pr > F
Model	12	16.46230000	1.37185833	1.83	0.0943
Error	27	20.27326000	0.75086148		
Corrected Total	39	36.73556000			

R-Square	Coeff Var	Root MSE	DMDmix Mean
0.448130	1.302767	0.866523	66.51400

Source	DF	Anova SS	Mean Square	F Value	Pr > F
Block	3	8.97054000	2.99018000	3.98	0.0180
Fertilizer	9	7.49176000	0.83241778	1.11	0.3899

The ANOVA Procedure

Dependent Variable: WSCmix

Source	DF	Sum of Squares	Mean Square	F Value	Pr > F
Model	12	8.72097000	0.72674750	0.64	0.7930
Error	27	30.83292750	1.14196028		
Corrected Total	39	39.55389750			

R-Square	Coeff Var	Root MSE	WSCmix Mean
0.220483	4.783516	1.068625	22.33975

Source	DF	Anova SS	Mean Square	F Value	Pr > F
Block	3	6.65114750	2.21704917	1.94	0.1467
Fertilizer	9	2.06982250	0.22998028	0.20	0.9919

The ANOVA Procedure

Dependent Variable: ADLmix

Source	DF	Sum of Squares	Mean Square	F Value	Pr > F
Model	12	1.20007000	0.10000583	1.77	0.1070
Error	27	1.52872750	0.05661954		
Corrected Total	39	2.72879750			

R-Square	Coeff Var	Root MSE	ADLmix Mean
0.439780	6.391743	0.237949	3.722750

Source	DF	Anova SS	Mean Square	F Value	Pr > F
Block	3	1.09284750	0.36428250	6.43	0.0020
Fertilizer	9	0.10722250	0.01191361	0.21	0.9905

The ANOVA Procedure

Dependent Variable: ProteinYmix

Source	DF	Sum of Squares	Mean Square	F Value	Pr > F
Model	12	9552636.280	796053.023	350.02	<.0001
Error	27	61405.658	2274.284		
Corrected Total	39	9614041.938			

R-Square	Coeff Var	Root MSE	ProteinYmix Mean
0.993613	3.567028	47.68945	1336.952

Source	DF	Anova SS	Mean Square	F Value	Pr > F
Block	3	29033.636	9677.879	4.26	0.0139
Fertilizer	9	9523602.644	1058178.072	465.28	<.0001

The ANOVA Procedure

Dependent Variable: CPsilo

Source	DF	Sum of Squares	Mean Square	F Value	Pr > F
Model	12	21.79692000	1.81641000	37.25	<.0001
Error	27	1.31672000	0.04876741		
Corrected Total	39	23.11364000			

R-Square	Coeff Var	Root MSE	CPsilo Mean
0.943033	1.750840	0.220833	12.61300

Source	DF	Anova SS	Mean Square	F Value	Pr > F
Block	3	0.44838000	0.14946000	3.06	0.0449
Fertilizer	9	21.34854000	2.37206000	48.64	<.0001

The ANOVA Procedure

Dependent Variable: NDFsilo

Source	DF	Sum of Squares	Mean Square	F Value	Pr > F
Model	12	638.7544200	53.2295350	31.76	<.0001
Error	27	45.2549800	1.6761104		
Corrected Total	39	684.0094000			

R-Square	Coeff Var	Root MSE	NDFsilo Mean
0.933839	2.606759	1.294647	49.66500

Source	DF	Anova SS	Mean Square	F Value	Pr > F
Block	3	3.0724200	1.0241400	0.61	0.6137
Fertilizer	9	635.6820000	70.6313333	42.14	<.0001

The ANOVA Procedure

Dependent Variable: ADFsilo

Source	DF	Sum of Squares	Mean Square	F Value	Pr > F
Model	12	697.0128900	58.0844075	36.52	<.0001
Error	27	42.9425075	1.5904632		
Corrected Total	39	739.9553975			

R-Square	Coeff Var	Root MSE	ADFsilo Mean
0.941966	4.627554	1.261136	27.25275

Source	DF	Anova SS	Mean Square	F Value	Pr > F

Block	3	2.7001675	0.9000558	0.57	0.6422
Fertilizer	9	694.3127225	77.1458581	48.51	<.0001

The ANOVA Procedure

Dependent Variable: DMDsilo

Source	DF	Sum of Squares	Mean Square	F Value	Pr > F
Model	12	3.17087000	0.26423917	0.42	0.9399
Error	27	16.82064000	0.62298667		
Corrected Total	39	19.99151000			

R-Square	Coeff Var	Root MSE	DMDsilo Mean
0.158611	1.349880	0.789295	58.47150

Source	DF	Anova SS	Mean Square	F Value	Pr > F
Block	3	0.08261000	0.02753667	0.04	0.9874
Fertilizer	9	3.08826000	0.34314000	0.55	0.8242

The ANOVA Procedure

Dependent Variable: WSCsilo

Source	DF	Sum of Squares	Mean Square	F Value	Pr > F
Model	12	2.60545000	0.21712083	0.29	0.9866
Error	27	20.44274000	0.75713852		
Corrected Total	39	23.04819000			

R-Square	Coeff Var	Root MSE	WSCsilo Mean
0.113044	4.946631	0.870137	17.59050

Source	DF	Anova SS	Mean Square	F Value	Pr > F
Block	3	1.26771000	0.42257000	0.56	0.6472
Fertilizer	9	1.33774000	0.14863778	0.20	0.9926

The ANOVA Procedure

Dependent Variable: ADLsilo

Source	DF	Sum of Squares	Mean Square	F Value	Pr > F
Model	12	0.39866000	0.03322167	0.82	0.6319
Error	27	1.09763750	0.04065324		

Corrected Total	39	1.49629750			

R-Square	Coeff Var	Root MSE	ADLsilo Mean
0.266431	5.844670	0.201626	3.449750

Source	DF	Anova SS	Mean Square	F Value	Pr > F
Block	3	0.14988750	0.04996250	1.23	0.3184
Fertilizer	9	0.24877250	0.02764139	0.68	0.7201

The ANOVA Procedure

Dependent Variable: CGRc

Source	DF	Sum of Squares	Mean Square	F Value	Pr > F
Model	12	523.0032000	43.5836000	91.43	<.0001
Error	27	12.8705100	0.4766856		
Corrected Total	39	535.8737100			

R-Square	Coeff Var	Root MSE	CGRc Mean
0.975982	2.735057	0.690424	25.24350

Source	DF	Anova SS	Mean Square	F Value	Pr > F
Block	3	0.3332900	0.1110967	0.23	0.8725
Fertilizer	9	522.6699100	58.0744344	121.83	<.0001

The ANOVA Procedure

Dependent Variable: CGRSs

Source	DF	Sum of Squares	Mean Square	F Value	Pr > F
Model	12	27.11303000	2.25941917	29.23	<.0001
Error	27	2.08676750	0.07728769		
Corrected Total	39	29.19979750			

R-Square	Coeff Var	Root MSE	CGRSs Mean
0.928535	3.281185	0.278007	8.472750

Source	DF	Anova SS	Mean Square	F Value	Pr > F
Block	3	1.66020750	0.55340250	7.16	0.0011
Fertilizer	9	25.45282250	2.82809139	36.59	<.0001

The ANOVA Procedure

Dependent Variable: LAIc

Source	DF	Sum of Squares	Mean Square	F Value	Pr > F
Model	12	1.93558000	0.16129833	150.19	<.0001
Error	27	0.02899750	0.00107398		
Corrected Total	39	1.96457750			

R-Square	Coeff Var	Root MSE	LAIc Mean
0.985240	1.428426	0.032772	2.294250

Source	DF	Anova SS	Mean Square	F Value	Pr > F
Block	3	0.00462750	0.00154250	1.44	0.2540
Fertilizer	9	1.93095250	0.21455028	199.77	<.0001

The ANOVA Procedure

Dependent Variable: LAIs

Source	DF	Sum of Squares	Mean Square	F Value	Pr > F
Model	12	1.86833000	0.15569417	110.43	<.0001
Error	27	0.03806750	0.00140991		
Corrected Total	39	1.90639750			

R-Square	Coeff Var	Root MSE	LAIs Mean
0.980032	1.270577	0.037549	2.955250

Source	DF	Anova SS	Mean Square	F Value	Pr > F
Block	3	0.00930750	0.00310250	2.20	0.1111
Fertilizer	9	1.85902250	0.20655806	146.50	<.0001

The ANOVA Procedure

Dependent Variable: PHc

Source	DF	Sum of Squares	Mean Square	F Value	Pr > F
Model	12	21508.50000	1792.37500	310.42	<.0001
Error	27	155.90000	5.77407		
Corrected Total	39	21664.40000			

R-Square	Coeff Var	Root MSE	PHc Mean
0.992804	1.391390	2.402930	172.7000

Source	DF	Anova SS	Mean Square	F Value	Pr > F
Block	3	20.60000	6.86667	1.19	0.3324
Fertilizer	9	21487.90000	2387.54444	413.49	<.0001

The ANOVA Procedure

Dependent Variable: PHs

Source	DF	Sum of Squares	Mean Square	F Value	Pr > F
Model	12	10867.80000	905.65000	395.67	<.0001
Error	27	61.80000	2.28889		
Corrected Total	39	10929.60000			

R-Square	Coeff Var	Root MSE	PHs Mean
0.994346	1.628533	1.512907	92.90000

Source	DF	Anova SS	Mean Square	F Value	Pr > F
Block	3	2.20000	0.73333	0.32	0.8105
Fertilizer	9	10865.60000	1207.28889	527.46	<.0001

The ANOVA Procedure

Dependent Variable: FYc

Source	DF	Sum of Squares	Mean Square	F Value	Pr > F
Model	12	1805.042950	150.420246	532.62	<.0001
Error	27	7.625247	0.282417		
Corrected Total	39	1812.668197			

R-Square	Coeff Var	Root MSE	FYc Mean
0.995793	2.188323	0.531429	24.28475

Source	DF	Anova SS	Mean Square	F Value	Pr > F
Block	3	0.324128	0.108043	0.38	0.7664
Fertilizer	9	1804.718822	200.524314	710.03	<.0001

The ANOVA Procedure

Dependent Variable: FYs

Source	DF	Sum of Squares	Mean Square	F Value	Pr > F

Model	12	1218.351910	101.529326	701.48	<.0001
Error	27	3.907890	0.144737		
Corrected Total	39	1222.259800			

R-Square	Coeff Var	Root MSE	FYs Mean
0.996803	2.314128	0.380443	16.44000

Source	DF	Anova SS	Mean Square	F Value	Pr > F
Block	3	1.091960	0.363987	2.51	0.0795
Fertilizer	9	1217.259950	135.251106	934.46	<.0001

The ANOVA Procedure

Dependent Variable: FYmix

Source	DF	Sum of Squares	Mean Square	F Value	Pr > F
Model	12	5941.814380	495.151198	1414.34	<.0001
Error	27	9.452530	0.350094		
Corrected Total	39	5951.266910			

R-Square	Coeff Var	Root MSE	FYmix Mean
0.998412	1.452938	0.591687	40.72350

Source	DF	Anova SS	Mean Square	F Value	Pr > F
Block	3	2.583870	0.861290	2.46	0.0843
Fertilizer	9	5939.230510	659.914501	1884.97	<.0001

The ANOVA Procedure

Dependent Variable: DMc

Source	DF	Sum of Squares	Mean Square	F Value	Pr > F
Model	12	110.9084300	9.2423692	168.21	<.0001
Error	27	1.4835475	0.0549462		
Corrected Total	39	112.3919775			

R-Square	Coeff Var	Root MSE	DMc Mean
0.986800	4.391066	0.234406	5.338250

Source	DF	Anova SS	Mean Square	F Value	Pr > F
Block	3	0.1495275	0.0498425	0.91	0.4506
Fertilizer	9	110.7589025	12.3065447	223.97	<.0001

The ANOVA Procedure

Dependent Variable: DMs

Source	DF	Sum of Squares	Mean Square	F Value	Pr > F
Model	12	81.73032000	6.81086000	191.24	<.0001
Error	27	0.96159000	0.03561444		
Corrected Total	39	82.69191000			

R-Square	Coeff Var	Root MSE	DMs Mean
0.988371	4.416003	0.188718	4.273500

Source	DF	Anova SS	Mean Square	F Value	Pr > F
Block	3	0.30881000	0.10293667	2.89	0.0537
Fertilizer	9	81.42151000	9.04683444	254.02	<.0001

The ANOVA Procedure

Dependent Variable: DMmix

Source	DF	Sum of Squares	Mean Square	F Value	Pr > F
Model	12	382.0224800	31.8352067	318.91	<.0001
Error	27	2.6952800	0.0998252		
Corrected Total	39	384.7177600			

R-Square	Coeff Var	Root MSE	DMmix Mean
0.992994	3.287392	0.315951	9.611000

Source	DF	Anova SS	Mean Square	F Value	Pr > F
Block	3	0.8637200	0.2879067	2.88	0.0541
Fertilizer	9	381.1587600	42.3509733	424.25	<.0001

The ANOVA Procedure

Dependent Variable: PH

Source	DF	Sum of Squares	Mean Square	F Value	Pr > F
Model	12	0.00690000	0.00057500	0.47	0.9169
Error	27	0.03326000	0.00123185		
Corrected Total	39	0.04016000			

R-Square	Coeff Var	Root MSE	PH Mean
0.171813	0.872210	0.035098	4.024000

Source	DF	Anova SS	Mean Square	F Value	Pr > F
Block	3	0.00614000	0.00204667	1.66	0.1988
Fertilizer	9	0.00076000	0.00008444	0.07	0.9999

The ANOVA Procedure

Dependent Variable: DM

Source	DF	Sum of Squares	Mean Square	F Value	Pr > F
Model	12	3.00037000	0.25003083	1.28	0.2857
Error	27	5.27650750	0.19542620		
Corrected Total	39	8.27687750			

R-Square	Coeff Var	Root MSE	DM Mean
0.362500	1.361023	0.442070	32.48075

Source	DF	Anova SS	Mean Square	F Value	Pr > F
Block	3	1.64186750	0.54728917	2.80	0.0590
Fertilizer	9	1.35850250	0.15094472	0.77	0.6425

The ANOVA Procedure

Dependent Variable: Lactic

Source	DF	Sum of Squares	Mean Square	F Value	Pr > F
Model	12	0.12503000	0.01041917	2.53	0.0222
Error	27	0.11133000	0.00412333		
Corrected Total	39	0.23636000			

R-Square	Coeff Var	Root MSE	Lactic Mean
0.528981	1.621955	0.064213	3.959000

Source	DF	Anova SS	Mean Square	F Value	Pr > F
Block	3	0.01202000	0.00400667	0.97	0.4205
Fertilizer	9	0.11301000	0.01255667	3.05	0.0120

The ANOVA Procedure

Dependent Variable: Acetic

Source	DF	Sum of Squares	Mean Square	F Value	Pr > F
Model	12	0.00392000	0.00032667	0.28	0.9877
Error	27	0.03135750	0.00116139		
Corrected Total	39	0.03527750			

R-Square	Coeff Var	Root MSE	Acetic Mean
0.111119	2.094923	0.034079	1.626750

Source	DF	Anova SS	Mean Square	F Value	Pr > F
Block	3	0.00276750	0.00092250	0.79	0.5078
Fertilizer	9	0.00115250	0.00012806	0.11	0.9992

The ANOVA Procedure

Dependent Variable: Propionic

Source	DF	Sum of Squares	Mean Square	F Value	Pr > F
Model	12	0.00410000	0.00034167	0.51	0.8907
Error	27	0.01813750	0.00067176		
Corrected Total	39	0.02223750			

R-Square	Coeff Var	Root MSE	Propionic Mean
0.184373	17.13608	0.025918	0.151250

Source	DF	Anova SS	Mean Square	F Value	Pr > F
Block	3	0.00168750	0.00056250	0.84	0.4852
Fertilizer	9	0.00241250	0.00026806	0.40	0.9246

The ANOVA Procedure

Dependent Variable: Butyric

Source	DF	Sum of Squares	Mean Square	F Value	Pr > F
Model	12	0.00085000	0.00007083	0.67	0.7627
Error	27	0.00284750	0.00010546		
Corrected Total	39	0.00369750			

R-Square	Coeff Var	Root MSE	Butyric Mean
0.229885	34.51938	0.010270	0.029750

Source	DF	Anova SS	Mean Square	F Value	Pr > F

Source	DF	Anova SS	Mean Square	F Value	Pr > F
Block	3	0.00052750	0.00017583	1.67	0.1975
Fertilizer	9	0.00032250	0.00003583	0.34	0.9532

The ANOVA Procedure

Dependent Variable: Total

Source	DF	Sum of Squares	Mean Square	F Value	Pr > F
Model	12	0.13486000	0.01123833	2.26	0.0387
Error	27	0.13447750	0.00498065		
Corrected Total	39	0.26933750			

R-Square	Coeff Var	Root MSE	Total Mean
0.500710	1.223910	0.070574	5.766250

Source	DF	Anova SS	Mean Square	F Value	Pr > F
Block	3	0.01084750	0.00361583	0.73	0.5454
Fertilizer	9	0.12401250	0.01377917	2.77	0.0196

The ANOVA Procedure

Dependent Variable: AmmoniaN

Source	DF	Sum of Squares	Mean Square	F Value	Pr > F
Model	12	0.62231000	0.05185917	2.96	0.0094
Error	27	0.47338750	0.01753287		
Corrected Total	39	1.09569750			

R-Square	Coeff Var	Root MSE	AmmoniaN Mean
0.567958	6.888373	0.132412	1.922250

Source	DF	Anova SS	Mean Square	F Value	Pr > F
Block	3	0.08498750	0.02832917	1.62	0.2089
Fertilizer	9	0.53732250	0.05970250	3.41	0.0065

The ANOVA Procedure

t Tests (LSD) for CPmix

NOTE: This test controls the Type I comparisonwise error rate, not the experimentwise error rate.

Alpha	0.05
Error Degrees of Freedom	27
Error Mean Square	0.050744
Critical Value of t	2.05183
Least Significant Difference	0.3268

Means with the same letter are not significantly different.

t Grouping	Mean	N	Fertilizer
A	14.5550	4	T5
A			
A	14.5500	4	T7
A			
A	14.5025	4	T1
A			
A	14.4775	4	T2
B	13.3825	4	T4
B			
B	13.3450	4	T6
B			
B	13.3325	4	T9
B			
B	13.3200	4	T3
B			
B	13.2825	4	T8
C	12.2800	4	T10

The ANOVA Procedure

t Tests (LSD) for NDFmix

NOTE: This test controls the Type I comparisonwise error rate, not the experimentwise error rate.

Alpha	0.05
Error Degrees of Freedom	27
Error Mean Square	1.281404
Critical Value of t	2.05183
Least Significant Difference	1.6424

Means with the same letter are not significantly different.

t Grouping	Mean	N	Fertilizer
A	58.5225	4	T6
A			
A	58.1300	4	T3
A			
A	58.0000	4	T4
A			
A	57.9300	4	T10
A			
A	57.7300	4	T9
A			

A	57.3900	4	T8
A			
A	57.3400	4	T1
B	49.6725	4	T5
B			
B	49.4550	4	T7
B			
B	49.2225	4	T2

The ANOVA Procedure

t Tests (LSD) for ADFmix

NOTE: This test controls the Type I comparisonwise error rate, not the experimentwise error rate.

Alpha	0.05
Error Degrees of Freedom	27
Error Mean Square	1.608891
Critical Value of t	2.05183
Least Significant Difference	1.8403

Means with the same letter are not significantly different.

t Grouping	Mean	N	Fertilizer
A	36.4800	4	T10
A			
A	36.1725	4	T4
A			
A	36.1225	4	T6
A			
A	36.0925	4	T3
A			
A	35.6800	4	T1
A			
A	35.2975	4	T9
A			
A	35.2250	4	T8
B	26.7900	4	T7
B			
B	26.7375	4	T5
B			
B	26.6550	4	T2

The ANOVA Procedure

t Tests (LSD) for DMDmix

NOTE: This test controls the Type I comparisonwise error rate, not the experimentwise error rate.

Alpha	0.05
Error Degrees of Freedom	27
Error Mean Square	0.750861
Critical Value of t	2.05183
Least Significant Difference	1.2572

Means with the same letter are not significantly different.

t Grouping	Mean	N	Fertilizer
A	66.9800	4	T4
A			
A	66.9500	4	T8
A			
A	66.9150	4	T9
A			
A	66.8175	4	T7
A			
A	66.7275	4	T5
A			
A	66.5250	4	T3
A			
A	66.3575	4	T10
A			
A	66.3125	4	T1
A			
A	65.8700	4	T6
A			
A	65.6850	4	T2

The ANOVA Procedure

t Tests (LSD) for WSCmix

NOTE: This test controls the Type I comparisonwise error rate, not the experimentwise error rate.

Alpha	0.05
Error Degrees of Freedom	27
Error Mean Square	1.14196
Critical Value of t	2.05183
Least Significant Difference	1.5504

Means with the same letter are not significantly different.

t Grouping	Mean	N	Fertilizer
A	22.7275	4	T7
A			
A	22.6975	4	T9
A			
A	22.4675	4	T6
A			
A	22.4275	4	T8
A			
A	22.3625	4	T3
A			
A	22.2125	4	T10
A			
A	22.1675	4	T4
A			
A	22.1625	4	T2
A			
A	22.1525	4	T5
A			
A	22.0200	4	T1

The ANOVA Procedure

t Tests (LSD) for ADLmix

NOTE: This test controls the Type I comparisonwise error rate, not the experimentwise error rate.

Alpha	0.05
Error Degrees of Freedom	27
Error Mean Square	0.05662
Critical Value of t	2.05183
Least Significant Difference	0.3452

Means with the same letter are not significantly different.

t Grouping	Mean	N	Fertilizer
A	3.7950	4	T8
A			
A	3.7725	4	T6
A			
A	3.7600	4	T10
A			
A	3.7475	4	T4
A			
A	3.7425	4	T3
A			
A	3.7400	4	T9
A			
A	3.7000	4	T7
A			
A	3.6875	4	T2
A			
A	3.6675	4	T1
A			
A	3.6150	4	T5

The ANOVA Procedure

t Tests (LSD) for ProteinYmix

NOTE: This test controls the Type I comparisonwise error rate, not the experimentwise error rate.

Alpha	0.05
Error Degrees of Freedom	27
Error Mean Square	2274.284
Critical Value of t	2.05183
Least Significant Difference	69.191

Means with the same letter are not significantly different.

t Grouping	Mean	N	Fertilizer
A	2009.98	4	T1
A			
A	2005.75	4	T7
A			
A	1991.95	4	T5
B	1408.95	4	T2
C	1307.87	4	T6
C			
C	1288.39	4	T9
D	923.74	4	T4

t Grouping	Mean	N	Fertilizer
D			
D	896.03	4	T8
D			
D	894.25	4	T3
E	642.62	4	T10

The ANOVA Procedure

t Tests (LSD) for CPsilo

NOTE: This test controls the Type I comparisonwise error rate, not the experimentwise error rate.

Alpha	0.05
Error Degrees of Freedom	27
Error Mean Square	0.048767
Critical Value of t	2.05183
Least Significant Difference	0.3204

Means with the same letter are not significantly different.

t Grouping	Mean	N	Fertilizer
A	13.4650	4	T5
A			
A	13.4325	4	T1
A			
A	13.4050	4	T7
A			
A	13.3850	4	T2
B	12.3525	4	T4
B			
B	12.2450	4	T6
B			
B	12.2325	4	T9
B			
B	12.2200	4	T3
B			
B	12.2125	4	T8
C	11.1800	4	T10

The ANOVA Procedure

t Tests (LSD) for NDFsilo

NOTE: This test controls the Type I comparisonwise error rate, not the experimentwise error rate.

Alpha	0.05
Error Degrees of Freedom	27
Error Mean Square	1.67611
Critical Value of t	2.05183
Least Significant Difference	1.8784

Means with the same letter are not significantly different.

t Grouping	Mean	N	Fertilizer

t Grouping	Mean	N	Fertilizer
A	52.8100	4	T6
A			
A	52.3075	4	T8
A			
A	52.2800	4	T3
A			
A	52.2275	4	T9
A			
A	52.1500	4	T4
A			
A	52.0900	4	T1
A			
A	52.0300	4	T10
B	43.7950	4	T5
B			
B	43.5975	4	T7
B			
B	43.3625	4	T2

The ANOVA Procedure

t Tests (LSD) for ADFsilo

NOTE: This test controls the Type I comparisonwise error rate, not the experimentwise error rate.

Alpha	0.05
Error Degrees of Freedom	27
Error Mean Square	1.590463
Critical Value of t	2.05183
Least Significant Difference	1.8297

Means with the same letter are not significantly different.

t Grouping	Mean	N	Fertilizer
A	30.5350	4	T10
A			
A	30.3275	4	T4
A			
A	30.2025	4	T3
A			
A	30.1700	4	T6
A			
A	29.7600	4	T1
A			
A	29.4425	4	T9
A			
A	29.3550	4	T8
B	20.9625	4	T5
B			
B	20.9450	4	T7
B			
B	20.8275	4	T2

The ANOVA Procedure

t Tests (LSD) for DMDsilo

NOTE: This test controls the Type I comparisonwise error rate, not the experimentwise error rate.

Alpha	0.05
Error Degrees of Freedom	27
Error Mean Square	0.622987
Critical Value of t	2.05183
Least Significant Difference	1.1452

Means with the same letter are not significantly different.

t Grouping	Mean	N	Fertilizer
A	58.9200	4	T9
A			
A	58.7425	4	T3
A			
A	58.7125	4	T5
A			
A	58.5925	4	T7
A			
A	58.5800	4	T10
A			
A	58.4525	4	T2
A			
A	58.3950	4	T8
A			
A	58.1750	4	T4
A			
A	58.0875	4	T6
A			
A	58.0575	4	T1

The ANOVA Procedure

t Tests (LSD) for WSCsilo

NOTE: This test controls the Type I comparisonwise error rate, not the experimentwise error rate.

Alpha	0.05
Error Degrees of Freedom	27
Error Mean Square	0.757139
Critical Value of t	2.05183
Least Significant Difference	1.2624

Means with the same letter are not significantly different.

t Grouping	Mean	N	Fertilizer
A	17.8150	4	T3
A			
A	17.8150	4	T8
A			
A	17.7625	4	T1
A			
A	17.7525	4	T7
A			
A	17.6225	4	T9
A			
A	17.6075	4	T4
A			
A	17.4250	4	T10
A			
A	17.3850	4	T5
A			

t Grouping	Mean	N	Fertilizer
A	17.3775	4	T2
A			
A	17.3425	4	T6

The ANOVA Procedure

t Tests (LSD) for ADLsilo

NOTE: This test controls the Type I comparisonwise error rate, not the experimentwise error rate.

Alpha	0.05
Error Degrees of Freedom	27
Error Mean Square	0.040653
Critical Value of t	2.05183
Least Significant Difference	0.2925

Means with the same letter are not significantly different.

t Grouping	Mean	N	Fertilizer
A	3.5400	4	T8
A			
A	3.5400	4	T6
A			
A	3.5325	4	T7
A			
A	3.5250	4	T10
A			
A	3.4500	4	T9
A			
A	3.4375	4	T3
A			
A	3.4250	4	T4
A			
A	3.3825	4	T5
A			
A	3.3500	4	T1
A			
A	3.3150	4	T2

The ANOVA Procedure

t Tests (LSD) for CGRc

NOTE: This test controls the Type I comparisonwise error rate, not the experimentwise error rate.

Alpha	0.05
Error Degrees of Freedom	27
Error Mean Square	0.476686
Critical Value of t	2.05183
Least Significant Difference	1.0017

Means with the same letter are not significantly different.

t Grouping	Mean	N	Fertilizer
A	29.3400	4	T1
A			
A	29.2300	4	T7

t Grouping	Mean	N	Fertilizer
A			
A	28.9800	4	T5
B	26.9775	4	T6
B			
B	26.3675	4	T2
B			
B	26.3450	4	T9
C	22.5100	4	T3
C			
C	22.4825	4	T8
C			
C	22.4000	4	T4
D	17.8025	4	T10

The ANOVA Procedure

t Tests (LSD) for CGRSs

NOTE: This test controls the Type I comparisonwise error rate, not the experimentwise error rate.

Alpha	0.05
Error Degrees of Freedom	27
Error Mean Square	0.077288
Critical Value of t	2.05183
Least Significant Difference	0.4033

Means with the same letter are not significantly different.

t Grouping	Mean	N	Fertilizer
A	9.6100	4	T7
A			
A	9.5500	4	T1
A			
A	9.3925	4	T5
B	8.6325	4	T6
B			
B	8.5475	4	T9
B			
B	8.4800	4	T2
C	7.7800	4	T8
C			
C	7.7525	4	T3
C			
C	7.7225	4	T4
D	7.2600	4	T10

The ANOVA Procedure

t Tests (LSD) for LAIc

NOTE: This test controls the Type I comparisonwise error rate, not the experimentwise error rate.

Alpha	0.05
Error Degrees of Freedom	27

Error Mean Square	0.001074
Critical Value of t	2.05183
Least Significant Difference	0.0475

Means with the same letter are not significantly different.

t Grouping	Mean	N	Fertilizer
A	2.55750	4	T1
A			
A	2.54250	4	T7
A			
A	2.51500	4	T5
B	2.38500	4	T2
B			
B	2.37000	4	T6
B			
B	2.34000	4	T9
C	2.15000	4	T3
C			
C	2.12250	4	T4
C			
C	2.12000	4	T8
D	1.84000	4	T10

The ANOVA Procedure

t Tests (LSD) for LAIs

NOTE: This test controls the Type I comparisonwise error rate, not the experimentwise error rate.

Alpha	0.05
Error Degrees of Freedom	27
Error Mean Square	0.00141
Critical Value of t	2.05183
Least Significant Difference	0.0545

Means with the same letter are not significantly different.

t Grouping	Mean	N	Fertilizer
A	3.20000	4	T7
A			
A	3.19750	4	T5
A			
A	3.16500	4	T1
B	3.07250	4	T2
B			
B	3.04500	4	T6
B			
B	3.02000	4	T9
C	2.78250	4	T3
C			
C	2.78000	4	T8
C			
C	2.75250	4	T4
D	2.53750	4	T10

The ANOVA Procedure

t Tests (LSD) for PHc

NOTE: This test controls the Type I comparisonwise error rate, not the experimentwise error rate.

Alpha	0.05
Error Degrees of Freedom	27
Error Mean Square	5.774074
Critical Value of t	2.05183
Least Significant Difference	3.4863

Means with the same letter are not significantly different.

t Grouping	Mean	N	Fertilizer
A	197.000	4	T1
A			
A	195.250	4	T5
A			
A	195.000	4	T7
B	186.250	4	T9
B			
B	185.000	4	T6
B			
B	184.000	4	T2
C	153.000	4	T8
C			
C	152.500	4	T3
C			
C	152.000	4	T4
D	127.000	4	T10

The ANOVA Procedure

t Tests (LSD) for PHs

NOTE: This test controls the Type I comparisonwise error rate, not the experimentwise error rate.

Alpha	0.05
Error Degrees of Freedom	27
Error Mean Square	2.288889
Critical Value of t	2.05183
Least Significant Difference	2.195

Means with the same letter are not significantly different.

t Grouping	Mean	N	Fertilizer
A	112.750	4	T5
A			
A	112.000	4	T7
B	109.250	4	T1

C	100.000	4	T6
C			
C	99.750	4	T9
C			
C	99.000	4	T2
D	78.000	4	T8
D			
D	77.750	4	T4
D			
D	76.500	4	T3
E	64.000	4	T10

The ANOVA Procedure

t Tests (LSD) for FYc

NOTE: This test controls the Type I comparisonwise error rate, not the experimentwise error rate.

Alpha	0.05
Error Degrees of Freedom	27
Error Mean Square	0.282417
Critical Value of t	2.05183
Least Significant Difference	0.771

Means with the same letter are not significantly different.

t Grouping	Mean	N	Fertilizer
A	33.3625	4	T5
A			
A	32.9000	4	T7
A			
A	32.6525	4	T1
B	25.4500	4	T6
B			
B	25.2025	4	T2
B			
B	25.1575	4	T9
C	18.2550	4	T4
C			
C	18.0225	4	T8
C			
C	17.6750	4	T3
D	14.1700	4	T10

The ANOVA Procedure

t Tests (LSD) for FYs

NOTE: This test controls the Type I comparisonwise error rate, not the experimentwise error rate.

Alpha	0.05
Error Degrees of Freedom	27
Error Mean Square	0.144737
Critical Value of t	2.05183
Least Significant Difference	0.552

Means with the same letter are not significantly different.

t Grouping	Mean	N	Fertilizer
A	24.3000	4	T5
A			
A	24.1775	4	T1
A			
A	24.1575	4	T7
B	15.8925	4	T9
B			
B	15.8475	4	T6
B			
B	15.6050	4	T2
C	12.3325	4	T4
D	11.7000	4	T3
D			
D	11.6750	4	T8
E	8.7125	4	T10

The ANOVA Procedure

t Tests (LSD) for FYmix

NOTE: This test controls the Type I comparisonwise error rate, not the experimentwise error rate.

Alpha	0.05
Error Degrees of Freedom	27
Error Mean Square	0.350094
Critical Value of t	2.05183
Least Significant Difference	0.8585

Means with the same letter are not significantly different.

t Grouping	Mean	N	Fertilizer
A	57.6625	4	T5
A			
A	57.0525	4	T7
A			
A	56.8275	4	T1
B	41.3000	4	T6
B			
B	41.0500	4	T9
B			
B	40.8025	4	T2
C	30.5875	4	T4
D	29.6975	4	T8
D			
D	29.3725	4	T3
E	22.8825	4	T10

The ANOVA Procedure

t Tests (LSD) for DMc

NOTE: This test controls the Type I comparisonwise error rate, not the experimentwise error rate.

Alpha	0.05
Error Degrees of Freedom	27
Error Mean Square	0.054946
Critical Value of t	2.05183
Least Significant Difference	0.3401

Means with the same letter are not significantly different.

t Grouping	Mean	N	Fertilizer
A	7.6700	4	T1
A			
A	7.5850	4	T7
A			
A	7.5025	4	T5
B	5.5250	4	T2
B			
B	5.3850	4	T6
B			
B	5.2975	4	T9
C	3.8575	4	T3
C			
C	3.8500	4	T8
C			
C	3.6775	4	T4
D	3.0325	4	T10

The ANOVA Procedure

t Tests (LSD) for DMs

NOTE: This test controls the Type I comparisonwise error rate, not the experimentwise error rate.

Alpha	0.05
Error Degrees of Freedom	27
Error Mean Square	0.035614
Critical Value of t	2.05183
Least Significant Difference	0.2738

Means with the same letter are not significantly different.

t Grouping	Mean	N	Fertilizer
A	6.2000	4	T7
A			
A	6.1900	4	T1
A			
A	6.1825	4	T5
B	4.4100	4	T6
B			
B	4.3650	4	T9

t Grouping	Mean	N	Fertilizer
B			
B	4.2075	4	T2
C	3.2225	4	T4
D	2.8975	4	T8
D			
D	2.8600	4	T3
E	2.2000	4	T10

The ANOVA Procedure

t Tests (LSD) for DMmix

NOTE: This test controls the Type I comparisonwise error rate, not the experimentwise error rate.

Alpha	0.05
Error Degrees of Freedom	27
Error Mean Square	0.099825
Critical Value of t	2.05183
Least Significant Difference	0.4584

Means with the same letter are not significantly different.

t Grouping	Mean	N	Fertilizer
A	13.8600	4	T1
A			
A	13.7875	4	T7
A			
A	13.6850	4	T5
B	9.7950	4	T6
B			
B	9.7300	4	T2
B			
B	9.6625	4	T9
C	6.9025	4	T4
C			
C	6.7450	4	T8
C			
C	6.7125	4	T3
D	5.2300	4	T10

The ANOVA Procedure

t Tests (LSD) for PH

NOTE: This test controls the Type I comparisonwise error rate, not the experimentwise error rate.

Alpha	0.05
Error Degrees of Freedom	27
Error Mean Square	0.001232
Critical Value of t	2.05183
Least Significant Difference	0.0509

Means with the same letter are not significantly different.

t Grouping	Mean	N	Fertilizer
A	4.03000	4	T2
A			
A	4.03000	4	T4
A			
A	4.02750	4	T10
A			
A	4.02750	4	T5
A			
A	4.02500	4	T6
A			
A	4.02250	4	T3
A			
A	4.02000	4	T1
A			
A	4.02000	4	T7
A			
A	4.02000	4	T8
A			
A	4.01750	4	T9

The ANOVA Procedure

t Tests (LSD) for DM

NOTE: This test controls the Type I comparisonwise error rate, not the experimentwise error rate.

Alpha	0.05
Error Degrees of Freedom	27
Error Mean Square	0.195426
Critical Value of t	2.05183
Least Significant Difference	0.6414

Means with the same letter are not significantly different.

t Grouping	Mean	N	Fertilizer
A	32.7800	4	T7
A			
A	32.7725	4	T5
A			
A	32.6050	4	T1
A			
A	32.5225	4	T3
A			
A	32.4900	4	T4
A			
A	32.4375	4	T10
A			
A	32.3875	4	T8
A			
A	32.3275	4	T6
A			
A	32.2500	4	T9
A			
A	32.2350	4	T2

The ANOVA Procedure

t Tests (LSD) for Lactic

NOTE: This test controls the Type I comparisonwise error rate, not the experimentwise error rate.

Alpha	0.05
Error Degrees of Freedom	27
Error Mean Square	0.004123
Critical Value of t	2.05183
Least Significant Difference	0.0932

Means with the same letter are not significantly different.

t Grouping			Mean	N	Fertilizer
	A		4.03500	4	T1
	A				
	A		4.02750	4	T7
	A				
B	A		4.01750	4	T2
B	A				
B	A	C	4.01250	4	T5
B		C			
B	D	C	3.92750	4	T6
	D	C			
	D	C	3.92000	4	T9
	D	C			
	D	C	3.92000	4	T4
	D	C			
	D	C	3.92000	4	T8
	D				
	D		3.91250	4	T3
	D				
	D		3.89750	4	T10

The ANOVA Procedure

t Tests (LSD) for Acetic

NOTE: This test controls the Type I comparisonwise error rate, not the experimentwise error rate.

Alpha	0.05
Error Degrees of Freedom	27
Error Mean Square	0.001161
Critical Value of t	2.05183
Least Significant Difference	0.0494

Means with the same letter are not significantly different.

t Grouping	Mean	N	Fertilizer
A	1.63500	4	T6
A			
A	1.63250	4	T10
A			
A	1.63250	4	T3
A			
A	1.63000	4	T7
A			
A	1.62750	4	T8
A			
A	1.62500	4	T4
A			

A	1.62500	4	T2
A			
A	1.62000	4	T5
A			
A	1.62000	4	T1
A			
A	1.62000	4	T9

The ANOVA Procedure

t Tests (LSD) for Propionic

NOTE: This test controls the Type I comparisonwise error rate, not the experimentwise error rate.

Alpha	0.05
Error Degrees of Freedom	27
Error Mean Square	0.000672
Critical Value of t	2.05183
Least Significant Difference	0.0376

Means with the same letter are not significantly different.

t Grouping	Mean	N	Fertilizer
A	0.16250	4	T5
A			
A	0.16000	4	T4
A			
A	0.15750	4	T2
A			
A	0.15750	4	T1
A			
A	0.15250	4	T3
A			
A	0.15000	4	T10
A			
A	0.14750	4	T9
A			
A	0.14500	4	T6
A			
A	0.14250	4	T7
A			
A	0.13750	4	T8

The ANOVA Procedure

t Tests (LSD) for Butyric

NOTE: This test controls the Type I comparisonwise error rate, not the experimentwise error rate.

Alpha	0.05
Error Degrees of Freedom	27
Error Mean Square	0.000105
Critical Value of t	2.05183
Least Significant Difference	0.0149

Means with the same letter are not significantly different.

t Grouping	Mean	N	Fertilizer

A	0.035000	4	T3
A			
A	0.032500	4	T1
A			
A	0.032500	4	T2
A			
A	0.030000	4	T10
A			
A	0.030000	4	T4
A			
A	0.030000	4	T5
A			
A	0.027500	4	T6
A			
A	0.027500	4	T7
A			
A	0.027500	4	T8
A			
A	0.025000	4	T9

The ANOVA Procedure

t Tests (LSD) for Total

NOTE: This test controls the Type I comparisonwise error rate, not the experimentwise error rate.

Alpha	0.05
Error Degrees of Freedom	27
Error Mean Square	0.004981
Critical Value of t	2.05183
Least Significant Difference	0.1024

Means with the same letter are not significantly different.

t Grouping		Mean	N	Fertilizer
	A	5.84750	4	T1
	A			
B	A	5.83250	4	T2
B	A			
B	A	5.82750	4	T7
B	A			
B	A	5.82500	4	T5
B				
B	C	5.73750	4	T4
B	C			
B	C	5.73750	4	T3
	C			
	C	5.72000	4	T9
	C			
	C	5.71750	4	T6
	C			
	C	5.71250	4	T8
	C			
	C	5.70500	4	T10

The ANOVA Procedure

t Tests (LSD) for AmmoniaN

NOTE: This test controls the Type I comparisonwise error rate, not the experimentwise error rate.

Alpha	0.05
Error Degrees of Freedom	27
Error Mean Square	0.017533
Critical Value of t	2.05183
Least Significant Difference	0.1921

Means with the same letter are not significantly different.

t Grouping	Mean	N	Fertilizer
A	2.08000	4	T5
A			
A	2.06500	4	T2
A			
A	2.06250	4	T1
A			
A	2.04750	4	T7
B	1.83750	4	T3
B			
B	1.83250	4	T9
B			
B	1.83000	4	T6
B			
B	1.83000	4	T4
B			
B	1.82250	4	T8
B			
B	1.81500	4	T10

--- Fertilizer=T1 ---

The MEANS Procedure

Variable	Mean	Mode	Median	Variance	Std Dev	Std Error
Block	2.5000000	.	2.5000000	1.6666667	1.2909944	0.6454972
CPmix	14.5025000	.	14.5000000	0.0088917	0.0942956	0.0471478
NDFmix	57.3400000	.	57.7200000	1.3264667	1.1517233	0.5758617
ADFmix	35.6800000	.	36.1600000	2.3265333	1.5252978	0.7626489
DMDmix	66.3125000	.	66.1000000	1.9110917	1.3824224	0.6912112
WSCmix	22.0200000	.	21.6400000	1.7254000	1.3135448	0.6567724
ADLmix	3.6675000	.	3.6800000	0.1042917	0.3229422	0.1614711
ProteinYmix	2009.98	.	2009.11	501.1786917	22.3870206	11.1935103
CPsilo	13.4325000	.	13.4300000	0.0088917	0.0942956	0.0471478
NDFsilo	52.0900000	.	52.3550000	2.8731333	1.6950320	0.8475160
ADFsilo	29.7600000	.	30.2700000	2.3719333	1.5401082	0.7700541
DMDsilo	58.0575000	.	57.4900000	1.5506917	1.2452677	0.6226339
WSCsilo	17.7625000	.	18.0750000	0.6952917	0.8338415	0.4169208

ADLsilo	3.3500000	.	3.3250000	0.0170000	0.1303840	0.0651920
CGRc	29.3400000	.	29.4550000	0.9004667	0.9489292	0.4744646
CGRSs	9.5500000	.	9.6700000	0.1638000	0.4047221	0.2023611
LAIc	2.5575000	2.5600000	2.5600000	0.0013583	0.0368556	0.0184278
LAIs	3.1650000	.	3.1700000	0.0017667	0.0420317	0.0210159
PHc	197.0000000	197.0000000	197.0000000	0.6666667	0.8164966	0.4082483
PHs	109.2500000	108.0000000	109.0000000	2.2500000	1.5000000	0.7500000
FYc	32.6525000	.	32.7450000	0.1384250	0.3720551	0.1860276
FYs	24.1775000	.	24.0250000	0.1673583	0.4090945	0.2045473
FYmix	56.8275000	.	56.8550000	0.4450917	0.6671519	0.3335760
DMc	7.6700000	.	7.5750000	0.0430000	0.2073644	0.1036822
DMs	6.1900000	.	6.2450000	0.0514000	0.2267157	0.1133578
DMmix	13.8600000	13.9400000	13.9050000	0.0139333	0.1180395	0.0590198
PH	4.0200000	4.0200000	4.0200000	0.0010667	0.0326599	0.0163299
DM	32.6050000	.	32.6450000	0.0111000	0.1053565	0.0526783
Lactic	4.0350000	.	4.0400000	0.0019667	0.0443471	0.0221736
Acetic	1.6200000	.	1.6200000	0.0011333	0.0336650	0.0168325
Propionic	0.1575000	.	0.1500000	0.0014917	0.0386221	0.0193111
Butyric	0.0325000	0.0300000	0.0300000	0.000158333	0.0125831	0.0062915
Total	5.8475000	.	5.8500000	0.0010917	0.0330404	0.0165202
AmmoniaN	2.0625000	1.9800000	1.9800000	0.0272250	0.1650000	0.0825000

--- Fertilizer=T10 ---

Variable	Mean	Mode	Median	Variance	Std Dev	Std Error
Block	2.5000000	.	2.5000000	1.6666667	1.2909944	0.6454972
CPmix	12.2800000	.	12.2950000	0.1051333	0.3242427	0.1621214
NDFmix	57.9300000	.	57.9450000	0.1224667	0.3499524	0.1749762
ADFmix	36.4800000	.	36.4800000	0.1083333	0.3291403	0.1645701
DMDmix	66.3575000	.	66.3700000	0.1282917	0.3581783	0.1790891
WSCmix	22.2125000	.	21.9950000	5.3272917	2.3080926	1.1540463

--- Fertilizer=T10 ---

The MEANS Procedure

Variable	Mean	Mode	Median	Variance	Std Dev	Std Error
ADLmix	3.7600000	.	3.7700000	0.0738000	0.2716616	0.1358308
ProteinYmix	642.6200000	.	629.9350000	1776.57	42.1493748	21.0746874
CPsilo	11.1800000	.	11.1950000	0.1051333	0.3242427	0.1621214
NDFsilo	52.0300000	.	52.0950000	0.1871333	0.4325891	0.2162946
ADFsilo	30.5350000	.	30.5650000	0.0568333	0.2383974	0.1191987
DMDsilo	58.5800000	.	58.5050000	0.0723333	0.2689486	0.1344743
WSCsilo	17.4250000	.	17.1600000	0.7899667	0.8888007	0.4444003
ADLsilo	3.5250000	.	3.5000000	0.0758333	0.2753785	0.1376893
CGRc	17.8025000	.	18.0950000	0.9934917	0.9967405	0.4983703
CGRSs	7.2600000	.	7.2800000	0.0224000	0.1496663	0.0748331
LAIc	1.8400000	1.8400000	1.8400000	0.000066667	0.0081650	0.0040825
LAIs	2.5375000	2.5400000	2.5400000	0.000025000	0.0050000	0.0025000
PHc	127.0000000	127.0000000	127.0000000	2.6666667	1.6329932	0.8164966
PHs	64.0000000	64.0000000	64.0000000	2.6666667	1.6329932	0.8164966
FYc	14.1700000	.	14.2400000	0.0780667	0.2794041	0.1397021
FYs	8.7125000	.	8.7500000	0.0397583	0.1993949	0.0996975
FYmix	22.8825000	.	23.0450000	0.2200250	0.4690682	0.2345341
DMc	3.0325000	3.0300000	3.0300000	0.0121583	0.1102648	0.0551324
DMs	2.2000000	.	2.1750000	0.0194000	0.1392839	0.0696419
DMmix	5.2300000	.	5.2050000	0.0592667	0.2434475	0.1217237
PH	4.0275000	4.0100000	4.0100000	0.0012250	0.0350000	0.0175000
DM	32.4375000	.	32.4700000	0.2646250	0.5144171	0.2572086
Lactic	3.8975000	.	3.9000000	0.000491667	0.0221736	0.0110868
Acetic	1.6325000	.	1.6250000	0.0011583	0.0340343	0.0170171
Propionic	0.1500000	.	0.1550000	0.0010000	0.0316228	0.0158114
Butyric	0.0300000	0.0300000	0.0300000	0.000066667	0.0081650	0.0040825
Total	5.7050000	.	5.7250000	0.0035000	0.0591608	0.0295804
AmmoniaN	1.8150000	1.8200000	1.8200000	0.000633333	0.0251661	0.0125831

-- Fertilizer=T2 --

Variable	Mean	Mode	Median	Variance	Std Dev	Std Error
Block	2.5000000	.	2.5000000	1.6666667	1.2909944	

						0.6454972
CPmix	14.4775000	.	14.5000000	0.0067583	0.0822091	0.0411045
NDFmix	49.2225000	.	49.1050000	0.1218250	0.3490344	0.1745172
ADFmix	26.6550000	.	26.6050000	0.2820333	0.5310681	0.2655341
DMDmix	65.6850000	.	65.7000000	0.1257667	0.3546360	0.1773180
WSCmix	22.1625000	.	22.2250000	0.5580250	0.7470107	0.3735054
ADLmix	3.6875000	.	3.7000000	0.1042917	0.3229422	0.1614711
ProteinYmix	1408.95	.	1412.22	2285.22	47.8039723	23.9019861
CPsilo	13.3850000	.	13.3800000	0.0291000	0.1705872	0.0852936
NDFsilo	43.3625000	.	43.2350000	0.1268250	0.3561250	0.1780625
ADFsilo	20.8275000	20.6800000	20.6800000	0.1923583	0.4385867	0.2192934
DMDsilo	58.4525000	.	58.3950000	0.5420250	0.7362235	0.3681117

--- Fertilizer=T2

The MEANS Procedure

Variable	Mean	Mode	Median	Variance	Std Dev	Std Error
WSCsilo	17.3775000	.	17.1450000	1.1988917	1.0949391	0.5474696
ADLsilo	3.3150000	.	3.2750000	0.0251000	0.1584298	0.0792149
CGRc	26.3675000	.	26.4300000	0.0329583	0.1815443	0.0907722
CGRSs	8.4800000	.	8.3850000	0.1948667	0.4414370	0.2207185
LAIc	2.3850000	2.3900000	2.3900000	0.0011000	0.0331662	0.0165831
LAIs	3.0725000	.	3.0600000	0.0036250	0.0602080	0.0301040
PHc	184.0000000	184.0000000	184.0000000	0.6666667	0.8164966	0.4082483
PHs	99.0000000	99.0000000	99.0000000	0.6666667	0.8164966	0.4082483
FYc	25.2025000	.	25.0400000	0.3390250	0.5822585	0.2911293
FYs	15.6050000	.	15.6900000	0.1371667	0.3703602	0.1851801
FYmix	40.8025000	.	40.7100000	0.4364917	0.6606752	0.3303376
DMc	5.5250000	.	5.5600000	0.0203667	0.1427118	0.0713559
DMs	4.2075000	.	4.2350000	0.0350250	0.1871497	0.0935748
DMmix	9.7300000	.	9.7350000	0.0868667	0.2947315	0.1473658
PH	4.0300000	3.9900000	4.0250000	0.0022000	0.0469042	0.0234521
DM	32.2350000	.	32.0150000	0.2359000	0.4856954	0.2428477
Lactic	4.0175000	3.9800000	4.0100000	0.0020250	0.0450000	0.0225000
Acetic	1.6250000	.	1.6400000	0.0027667	0.0525991	

0.0262996

Variable	Mean	Mode	Median	Variance	Std Dev	Std Error
Propionic	0.1575000	0.1700000	0.1650000	0.000358333	0.0189297	0.0094648
Butyric	0.0325000	0.0300000	0.0300000	0.000158333	0.0125831	0.0062915
Total	5.8325000	.	5.8150000	0.0038250	0.0618466	0.0309233
AmmoniaN	2.0650000	2.1100000	2.0900000	0.0043667	0.0660808	0.0330404

-- Fertilizer=T3 --

Variable	Mean	Mode	Median	Variance	Std Dev	Std Error
Block	2.5000000	.	2.5000000	1.6666667	1.2909944	0.6454972
CPmix	13.3200000	.	13.3150000	0.0822000	0.2867054	0.1433527
NDFmix	58.1300000	.	57.7600000	2.3036667	1.5177835	0.7588917
ADFmix	36.0925000	.	35.8550000	1.9942250	1.4121703	0.7060852
DMDmix	66.5250000	.	66.5400000	0.1336333	0.3655589	0.1827795
WSCmix	22.3625000	.	22.3750000	0.1044250	0.3231486	0.1615743
ADLmix	3.7425000	.	3.8150000	0.0914250	0.3023657	0.1511828
ProteinYmix	894.2450000	.	895.8250000	2794.41	52.8621847	26.4310923
CPsilo	12.2200000	.	12.2150000	0.0822000	0.2867054	0.1433527
NDFsilo	52.2800000	.	51.9100000	2.3036667	1.5177835	0.7588917
ADFsilo	30.2025000	.	29.9500000	1.9802917	1.4072284	0.7036142
DMDsilo	58.7425000	.	58.6650000	0.0717583	0.2678775	0.1339387
WSCsilo	17.8150000	.	17.7600000	1.2163000	1.1028599	0.5514300
ADLsilo	3.4375000	.	3.4050000	0.0288917	0.1699755	0.0849877
CGRc	22.5100000	.	22.5150000	0.0014000	0.0374166	0.0187083
CGRSs	7.7525000	.	7.7450000	0.0017583	0.0419325	0.0209662
LAIc	2.1500000	2.1800000	2.1650000	0.0018000	0.0424264	0.0212132
LAIs	2.7825000	.	2.7850000	0.0020917	0.0457347	0.0228674

-- Fertilizer=T3 --

The MEANS Procedure

Variable	Mean	Mode	Median	Variance	Std Dev	Std Error
PHc	152.5000000	153.0000000	153.0000000	6.3333333	2.5166115	1.2583057
PHs	76.5000000	.	76.5000000	1.6666667	1.2909944	0.6454972

FYc	17.6750000	.	17.7400000	0.0815000	0.2854820	0.1427410
FYs	11.7000000	.	11.7400000	0.0384667	0.1961292	0.0980646
FYmix	29.3725000	.	29.4750000	0.2304250	0.4800260	0.2400130
DMc	3.8575000	.	3.8400000	0.0640917	0.2531633	0.1265817
DMs	2.8600000	.	2.8350000	0.0130000	0.1140175	0.0570088
DMmix	6.7125000	.	6.6200000	0.1208250	0.3475989	0.1737995
PH	4.0225000	4.0100000	4.0100000	0.0015583	0.0394757	0.0197379
DM	32.5225000	.	32.6950000	0.3442917	0.5867637	0.2933819
Lactic	3.9125000	3.9600000	3.9250000	0.0034250	0.0585235	0.0292617
Acetic	1.6325000	.	1.6350000	0.000291667	0.0170783	0.0085391
Propionic	0.1525000	.	0.1550000	0.0010917	0.0330404	0.0165202
Butyric	0.0350000	.	0.0350000	0.000166667	0.0129099	0.0064550
Total	5.7375000	.	5.7350000	0.0016917	0.0411299	0.0205649
AmmoniaN	1.8375000	.	1.8400000	0.000491667	0.0221736	0.0110868

ƒƒ

--- Fertilizer=T4 ---

Variable	Mean	Mode	Median	Variance	Std Dev	Std Error
Block	2.5000000	.	2.5000000	1.6666667	1.2909944	0.6454972
CPmix	13.3825000	.	13.2850000	0.0479583	0.2189939	0.1094970
NDFmix	58.0000000	.	58.0150000	2.2164667	1.4887803	0.7443901
ADFmix	36.1725000	.	36.7750000	1.7794250	1.3339509	0.6669754
DMDmix	66.9800000	.	66.9950000	0.1247333	0.3531761	0.1765880
WSCmix	22.1675000	.	22.1800000	0.1066250	0.3265348	0.1632674
ADLmix	3.7475000	.	3.7600000	0.1042917	0.3229422	0.1614711
ProteinYmix	923.7400000	.	946.0300000	2717.13	52.1260543	26.0630271
CPsilo	12.3525000	.	12.2550000	0.0479583	0.2189939	0.1094970
NDFsilo	52.1500000	.	52.1650000	2.2164667	1.4887803	0.7443901
ADFsilo	30.3275000	.	30.9450000	1.8478250	1.3593473	0.6796736
DMDsilo	58.1750000	.	58.2650000	0.8696333	0.9325413	0.4662707
WSCsilo	17.6075000	.	17.6600000	0.0411583	0.2028752	0.1014376
ADLsilo	3.4250000	.	3.4450000	0.0635000	0.2519921	0.1259960
CGRc	22.4000000	.	22.4150000	0.0016667	0.0408248	0.0204124
CGRSs	7.7225000	.	7.7250000	0.0012250	0.0350000	0.0175000
LAIc	2.1225000	2.1200000	2.1200000	0.000158333	0.0125831	0.0062915

LAIs	2.7525000	.	2.7600000	0.000958333	0.0309570	0.0154785
PHc	152.0000000	152.0000000	152.0000000	0.6666667	0.8164966	0.4082483
PHs	77.7500000	79.0000000	78.0000000	2.2500000	1.5000000	0.7500000
FYc	18.2550000	.	18.2550000	0.0532333	0.2307235	0.1153617
FYs	12.3325000	.	12.3400000	0.0454917	0.2132878	0.1066439
FYmix	30.5875000	.	30.6500000	0.0552250	0.2350000	0.1175000
DMc	3.6775000	.	3.7300000	0.0635583	0.2521078	0.1260539

-- Fertilizer=T4 --

The MEANS Procedure

Variable	Mean	Mode	Median	Variance	Std Dev	Std Error
DMs	3.2225000	.	3.2450000	0.0251583	0.1586138	0.0793069
DMmix	6.9025000	.	7.0450000	0.1312917	0.3623419	0.1811710
PH	4.0300000	4.0600000	4.0350000	0.0012667	0.0355903	0.0177951
DM	32.4900000	.	32.6550000	0.2200667	0.4691126	0.2345563
Lactic	3.9200000	.	3.9000000	0.0074667	0.0864099	0.0432049
Acetic	1.6250000	.	1.6250000	0.000433333	0.0208167	0.0104083
Propionic	0.1600000	0.1500000	0.1500000	0.000733333	0.0270801	0.0135401
Butyric	0.0300000	0.0400000	0.0350000	0.000200000	0.0141421	0.0070711
Total	5.7375000	.	5.7400000	0.0048917	0.0699405	0.0349702
AmmoniaN	1.8300000	1.8800000	1.8750000	0.0087333	0.0934523	0.0467262

-- Fertilizer=T5 --

Variable	Mean	Mode	Median	Variance	Std Dev	Std Error
Block	2.5000000	.	2.5000000	1.6666667	1.2909944	0.6454972
CPmix	14.5550000	.	14.5600000	0.0081667	0.0903696	0.0451848
NDFmix	49.6725000	.	49.6500000	0.4927583	0.7019675	0.3509837
ADFmix	26.7375000	.	26.7500000	0.1084917	0.3293807	0.1646904
DMDmix	66.7275000	.	66.5200000	6.3016250	2.5103038	1.2551519
WSCmix	22.1525000	.	21.9500000	0.2675583	0.5172604	0.2586302
ADLmix	3.6150000	.	3.6300000	0.1017667	0.3190089	0.1595044
ProteinYmix	1991.95	.	1993.02	628.0695000	25.0613148	

12.5306574

Variable	Mean	Mode	Median	Variance	Std Dev	Std Error
CPsilo	13.4650000	.	13.4700000	0.0081667	0.0903696	0.0451848
NDFsilo	43.7950000	.	43.8000000	0.4577000	0.6765353	0.3382676
ADFsilo	20.9625000	.	20.8800000	0.2493583	0.4993579	0.2496790
DMDsilo	58.7125000	.	58.7800000	0.0732917	0.2707243	0.1353622
WSCsilo	17.3850000	.	17.5400000	0.4931667	0.7022583	0.3511291
ADLsilo	3.3825000	.	3.4300000	0.0250917	0.1584035	0.0792017
CGRc	28.9800000	.	28.8400000	0.5120667	0.7155883	0.3577942
CGRSs	9.3925000	.	9.6000000	0.3794917	0.6160289	0.3080145
LAIc	2.5150000	.	2.5100000	0.0043667	0.0660808	0.0330404
LAIs	3.1975000	.	3.1850000	0.0034917	0.0590903	0.0295452
PHc	195.2500000	198.0000000	198.0000000	38.2500000	6.1846584	3.0923292
PHs	112.7500000	.	113.5000000	6.9166667	2.6299556	1.3149778
FYc	33.3625000	.	33.0900000	1.0516250	1.0254877	0.5127438
FYs	24.3000000	.	23.9250000	0.9444667	0.9718367	0.4859184
FYmix	57.6625000	.	57.7300000	1.4089583	1.1869955	0.5934978
DMc	7.5025000	.	7.5150000	0.1068917	0.3269429	0.1634715
DMs	6.1825000	.	6.1050000	0.0784250	0.2800446	0.1400223
DMmix	13.6850000	.	13.7500000	0.0515667	0.2270830	0.1135415
PH	4.0275000	4.0100000	4.0100000	0.0012250	0.0350000	0.0175000
DM	32.7725000	.	32.7200000	0.0361583	0.1901534	0.0950767
Lactic	4.0125000	.	4.0000000	0.0024917	0.0499166	0.0249583
Acetic	1.6200000	.	1.6150000	0.0020667	0.0454606	0.0227303

-- Fertilizer=T5 --

The MEANS Procedure

Variable	Mean	Mode	Median	Variance	Std Dev	Std Error
Propionic	0.1625000	0.1600000	0.1600000	0.000425000	0.0206155	0.0103078
Butyric	0.0300000	0.0300000	0.0300000	0.000066667	0.0081650	0.0040825
Total	5.8250000	.	5.8400000	0.0056333	0.0750555	0.0375278
AmmoniaN	2.0800000	.	2.0800000	0.0150667	0.1227464	0.0613732

-- Fertilizer=T6 --

Variable	Mean	Mode	Median	Variance	Std Dev	Std Error
Block	2.5000000	.	2.5000000	1.6666667	1.2909944	0.6454972
CPmix	13.3450000	.	13.3400000	0.0321000	0.1791647	0.0895824
NDFmix	58.5225000	.	59.0400000	2.9239583	1.7099586	0.8549793
ADFmix	36.1225000	.	36.2150000	2.0992917	1.4488933	0.7244466
DMDmix	65.8700000	.	65.8850000	0.1296667	0.3600926	0.1800463
WSCmix	22.4675000	.	22.8350000	0.8021583	0.8956329	0.4478165
ADLmix	3.7725000	3.8200000	3.8200000	0.0778250	0.2789713	0.1394857
ProteinYmix	1307.87	.	1270.81	12669.37	112.5583028	56.2791514
CPsilo	12.2450000	.	12.2400000	0.0321000	0.1791647	0.0895824
NDFsilo	52.8100000	.	53.1900000	2.2336667	1.4945456	0.7472728
ADFsilo	30.1700000	.	30.3850000	1.7850000	1.3360389	0.6680195
DMDsilo	58.0875000	.	58.0050000	0.0779583	0.2792102	0.1396051
WSCsilo	17.3425000	.	17.3300000	0.3452917	0.5876152	0.2938076
ADLsilo	3.5400000	.	3.6150000	0.0579333	0.2406934	0.1203467
CGRc	26.9775000	.	26.9850000	0.0817583	0.2859341	0.1429671
CGRSs	8.6325000	.	8.6400000	0.2054917	0.4533119	0.2266559
LAIc	2.3700000	2.3700000	2.3700000	0.000266667	0.0163299	0.0081650
LAIs	3.0450000	3.0700000	3.0500000	0.000900000	0.0300000	0.0150000
PHc	185.0000000	185.0000000	185.0000000	0.6666667	0.8164966	0.4082483
PHs	100.0000000	100.0000000	100.0000000	0.6666667	0.8164966	0.4082483
FYc	25.4500000	25.8600000	25.4550000	0.2242000	0.4734976	0.2367488
FYs	15.8475000	.	15.8100000	0.1222917	0.3497023	0.1748511
FYmix	41.3000000	.	41.2550000	0.4443333	0.6665833	0.3332917
DMc	5.3850000	.	5.2350000	0.1341667	0.3662877	0.1831438
DMs	4.4100000	.	4.3650000	0.1448667	0.3806135	0.1903068
DMmix	9.7950000	.	9.5600000	0.5191000	0.7204859	0.3602430
PH	4.0250000	3.9800000	4.0200000	0.0027667	0.0525991	0.0262996
DM	32.3275000	.	32.3900000	0.1709583	0.4134711	0.2067355
Lactic	3.9275000	.	3.9300000	0.0030917	0.0556028	0.0278014
Acetic	1.6350000	1.6100000	1.6150000	0.0019000	0.0435890	0.0217945
Propionic	0.1450000	.	0.1400000	0.000700000	0.0264575	0.0132288
Butyric	0.0275000	0.0300000	0.0300000	0.000025000	0.0050000	0.0025000
Total	5.7175000	.	5.7050000	0.0096917	0.0984463	0.0492231
AmmoniaN	1.8300000	.	1.8350000	0.0098000	0.0989949	

0.0494975

--- Fertilizer=T7 ---

The MEANS Procedure

Variable	Mean	Mode	Median	Variance	Std Dev	Std Error
Block	2.5000000	.	2.5000000	1.6666667	1.2909944	0.6454972
CPmix	14.5500000	.	14.5500000	0.0194667	0.1395230	0.0697615
NDFmix	49.4550000	.	49.4700000	0.1147667	0.3387723	0.1693861
ADFmix	26.7900000	.	26.8050000	0.1080667	0.3287349	0.1643675
DMDmix	66.8175000	.	66.8250000	0.1191583	0.3451932	0.1725966
WSCmix	22.7275000	.	22.7350000	0.1149583	0.3390551	0.1695275
ADLmix	3.7000000	3.7200000	3.7200000	0.1019333	0.3192700	0.1596350
ProteinYmix	2005.75	.	2001.51	2246.79	47.4002844	23.7001422
CPsilo	13.4050000	13.3900000	13.3900000	0.0019667	0.0443471	0.0221736
NDFsilo	43.5975000	.	43.6200000	0.1230917	0.3508442	0.1754221
ADFsilo	20.9450000	.	20.9500000	0.0888333	0.2980492	0.1490246
DMDsilo	58.5925000	.	58.9200000	0.5984250	0.7735793	0.3867897
WSCsilo	17.7525000	.	17.8300000	0.5715583	0.7560148	0.3780074
ADLsilo	3.5325000	.	3.5800000	0.0250917	0.1584035	0.0792017
CGRc	29.2300000	.	29.3050000	0.8700667	0.9327736	0.4663868
CGRSs	9.6100000	.	9.7400000	0.1938667	0.4403029	0.2201515
LAIc	2.5425000	2.5400000	2.5400000	0.0013583	0.0368556	0.0184278
LAIs	3.2000000	3.2000000	3.2000000	0.000600000	0.0244949	0.0122474
PHc	195.0000000	195.0000000	195.0000000	0.6666667	0.8164966	0.4082483
PHs	112.0000000	112.0000000	112.0000000	0.6666667	0.8164966	0.4082483
FYc	32.9000000	.	33.0150000	0.1138000	0.3373426	0.1686713
FYs	24.1575000	.	24.1700000	0.0184250	0.1357387	0.0678694
FYmix	57.0525000	.	57.1750000	0.2160917	0.4648566	0.2324283
DMc	7.5850000	.	7.5600000	0.0575000	0.2397916	0.1198958
DMs	6.2000000	.	6.2400000	0.0230667	0.1518771	0.0759386
DMmix	13.7875000	.	13.7000000	0.0996250	0.3156343	0.1578171
PH	4.0200000	3.9900000	4.0050000	0.0018000	0.0424264	0.0212132
DM	32.7800000	.	32.9150000	0.1692667	0.4114203	0.2057102
Lactic	4.0275000	4.0100000	4.0100000	0.0012250	0.0350000	

						0.0175000
Acetic	1.6300000	1.6300000	1.6300000	0.000066667	0.0081650	0.0040825
Propionic	0.1425000	0.1400000	0.1400000	0.000158333	0.0125831	0.0062915
Butyric	0.0275000	0.0300000	0.0300000	0.000158333	0.0125831	0.0062915
Total	5.8275000	5.8100000	5.8100000	0.0024250	0.0492443	0.0246221
AmmoniaN	2.0475000	.	2.0550000	0.0098250	0.0991211	0.0495606

--- Fertilizer=T8 ---

Variable	Mean	Mode	Median	Variance	Std Dev	Std Error
Block	2.5000000	.	2.5000000	1.6666667	1.2909944	0.6454972
CPmix	13.2825000	.	13.1800000	0.1630917	0.4038461	0.2019230
NDFmix	57.3900000	.	57.4050000	0.1224667	0.3499524	0.1749762
ADFmix	35.2250000	.	34.9750000	5.5221667	2.3499291	1.1749645
DMDmix	66.9500000	.	67.2150000	0.6530000	0.8080842	0.4040421
WSCmix	22.4275000	.	22.1650000	0.5582917	0.7471892	0.3735946

--- Fertilizer=T8 ---

The MEANS Procedure

Variable	Mean	Mode	Median	Variance	Std Dev	Std Error
ADLmix	3.7950000	.	3.8950000	0.0472333	0.2173323	0.1086662
ProteinYmix	896.0325000	.	896.2900000	1168.50	34.1833365	17.0916682
CPsilo	12.2125000	.	12.1100000	0.1630917	0.4038461	0.2019230
NDFsilo	52.3075000	.	52.0550000	1.3928917	1.1802083	0.5901042
ADFsilo	29.3550000	.	29.1050000	5.5221667	2.3499291	1.1749645
DMDsilo	58.3950000	.	58.6900000	1.3790333	1.1743225	0.5871612
WSCsilo	17.8150000	.	17.8050000	1.8509667	1.3605024	0.6802512
ADLsilo	3.5400000	.	3.5550000	0.0624667	0.2499333	0.1249667
CGRc	22.4825000	.	22.6000000	0.8273583	0.9095924	0.4547962
CGRSs	7.7800000	.	7.7050000	0.0816667	0.2857738	0.1428869
LAIc	2.1200000	2.1200000	2.1200000	0.000266667	0.0163299	0.0081650
LAIs	2.7800000	2.7800000	2.7800000	0.000066667	0.0081650	0.0040825
PHc	153.0000000	153.0000000	153.0000000	2.6666667	1.6329932	0.8164966

PHs	78.0000000	78.0000000	78.0000000	0.6666667	0.8164966	0.4082483
FYc	18.0225000	.	18.0200000	0.0522250	0.2285279	0.1142639
FYs	11.6750000	11.7800000	11.7700000	0.0387667	0.1968925	0.0984463
FYmix	29.6975000	.	29.6900000	0.0184917	0.1359841	0.0679920
DMc	3.8500000	3.8700000	3.8600000	0.000800000	0.0282843	0.0141421
DMs	2.8975000	.	2.8800000	0.0146917	0.1212092	0.0606046
DMmix	6.7450000	.	6.7350000	0.0089667	0.0946925	0.0473462
PH	4.0200000	4.0200000	4.0200000	0	0	0
DM	32.3875000	.	32.2850000	0.5721583	0.7564115	0.3782057
Lactic	3.9200000	.	3.9350000	0.0138000	0.1174734	0.0587367
Acetic	1.6275000	.	1.6300000	0.0010917	0.0330404	0.0165202
Propionic	0.1375000	.	0.1350000	0.000625000	0.0250000	0.0125000
Butyric	0.0275000	0.0300000	0.0300000	0.000025000	0.0050000	0.0025000
Total	5.7125000	.	5.7400000	0.0108917	0.1043631	0.0521816
AmmoniaN	1.8225000	.	1.7150000	0.1086917	0.3296842	0.1648421

ƒƒ

--- Fertilizer=T9 --

Variable	Mean	Mode	Median	Variance	Std Dev	Std Error
Block	2.5000000	.	2.5000000	1.6666667	1.2909944	0.6454972
CPmix	13.3325000	.	13.3250000	0.1097583	0.3312980	0.1656490
NDFmix	57.7300000	.	57.7150000	3.3900667	1.8412134	0.9206067
ADFmix	35.2975000	.	35.1500000	1.1342250	1.0650000	0.5325000
DMDmix	66.9150000	.	66.9300000	0.1209667	0.3478026	0.1739013
WSCmix	22.6975000	.	23.1850000	2.9299583	1.7117121	0.8558561
ADLmix	3.7400000	.	3.7200000	0.0670000	0.2588436	0.1294218
ProteinYmix	1288.39	.	1280.73	3359.20	57.9585929	28.9792965
CPsilo	12.2325000	.	12.2250000	0.1097583	0.3312980	0.1656490
NDFsilo	52.2275000	.	52.5200000	4.1945583	2.0480621	1.0240310
ADFsilo	29.4425000	.	29.3100000	1.1196250	1.0581233	0.5290617
DMDsilo	58.9200000	.	59.0600000	0.3992667	0.6318755	0.3159378

ƒƒ

--- Fertilizer=T9 --

The MEANS Procedure

Variable	Mean	Mode	Median	Variance	Std Dev	Std Error
WSCsilo	17.6225000	.	17.6400000	0.0342250	0.1850000	0.0925000
ADLsilo	3.4500000	.	3.4600000	0.0349333	0.1869046	0.0934523
CGRc	26.3450000	25.9800000	26.3150000	0.1800333	0.4243034	0.2121517
CGRSs	8.5475000	.	8.5300000	0.0044250	0.0665207	0.0332603
LAIc	2.3400000	.	2.3350000	0.000466667	0.0216025	0.0108012
LAIs	3.0200000	.	3.0200000	0.0022667	0.0476095	0.0238048
PHc	186.2500000	188.0000000	187.0000000	5.5833333	2.3629078	1.1814539
PHs	99.7500000	.	99.5000000	2.9166667	1.7078251	0.8539126
FYc	25.1575000	.	25.1850000	0.5176917	0.7195079	0.3597540
FYs	15.8925000	.	15.9200000	0.1144250	0.3382676	0.1691338
FYmix	41.0500000	.	41.3600000	0.5370000	0.7328028	0.3664014
DMc	5.2975000	.	5.3300000	0.0418250	0.2045116	0.1022558
DMs	4.3650000	.	4.3500000	0.0184333	0.1357694	0.0678847
DMmix	9.6625000	.	9.6650000	0.0948917	0.3080449	0.1540225
PH	4.0175000	4.0200000	4.0200000	0.000025000	0.0050000	0.0025000
DM	32.2500000	32.3300000	32.3300000	0.2816000	0.5306600	0.2653300
Lactic	3.9200000	.	3.9250000	0.0051333	0.0716473	0.0358236
Acetic	1.6200000	.	1.6250000	0.000466667	0.0216025	0.0108012
Propionic	0.1475000	0.1500000	0.1500000	0.000025000	0.0050000	0.0025000
Butyric	0.0250000	0.0300000	0.0300000	0.000100000	0.0100000	0.0050000
Total	5.7200000	5.6600000	5.7200000	0.0048000	0.0692820	0.0346410
AmmoniaN	1.8325000	.	1.8450000	0.0012917	0.0359398	0.0179699
