# Supplementary figures and images for: Impact of organic and inorganic fertilizers on the yield and quality of silage corn intercropped with soybean
Source: PeerJ. 2018 Oct 26;6:e5280. doi: 10.7717/peerj.5280 (PMC6204818; doi:10.7717/peerj.5280)

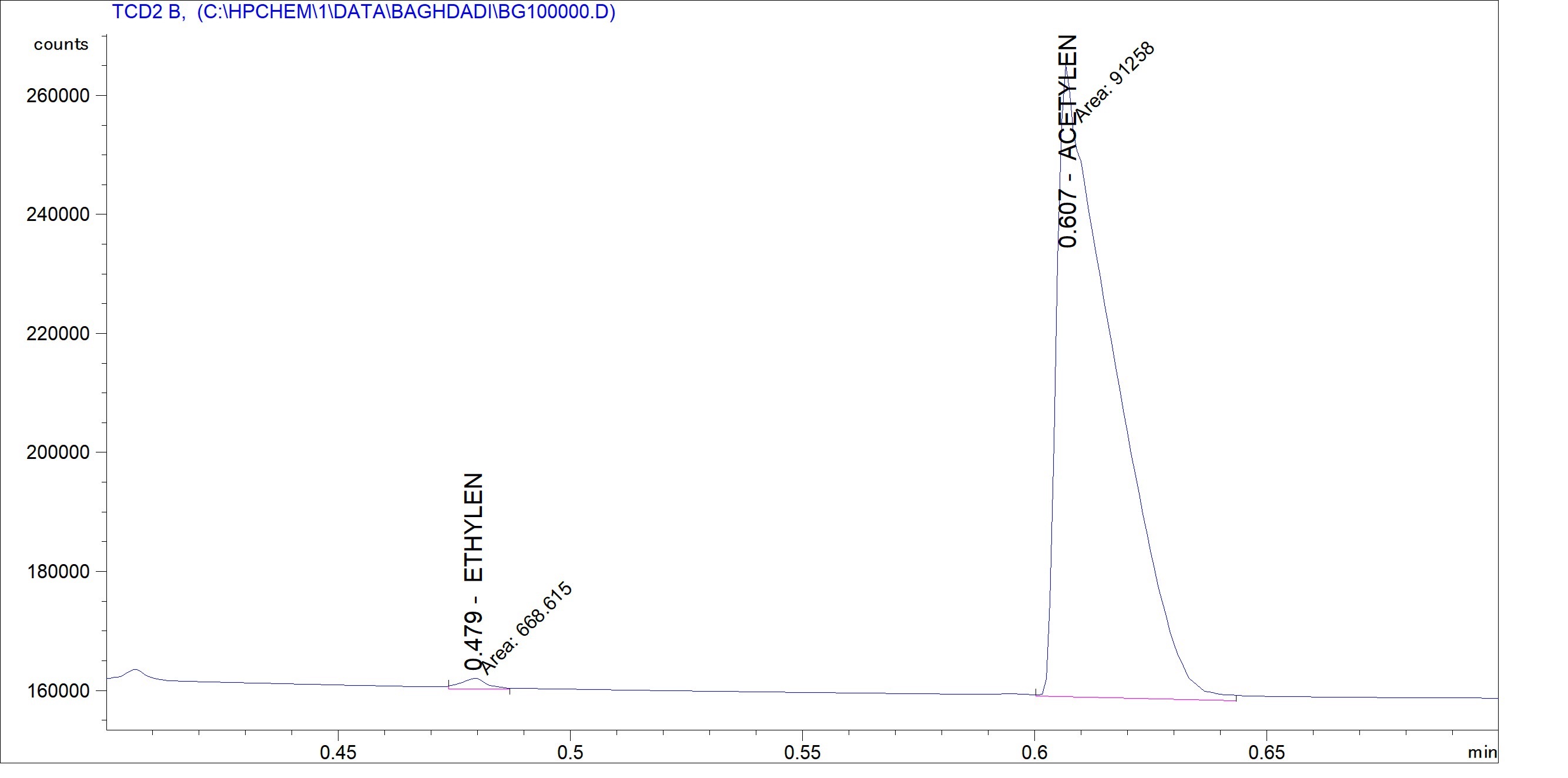

Supplement: Supplemental Information 5 [file peerj-06-5280-s005.jpg]
